# A flexible method for aggregation of prior statistical findings

**DOI:** 10.1371/journal.pone.0175111

**Published:** 2017-04-06

**Authors:** Hazhir Rahmandad, Mohammad S. Jalali, Kamran Paynabar

**Affiliations:** 1 Sloan School of Management, Massachusetts Institute of Technology, Cambridge, Massachusetts, United States of America; 2 Industrial and Systems Engineering, Georgia Institute of Technology, Atlanta, Georgia, United States of America; Brown University, UNITED STATES

## Abstract

Rapid growth in scientific output requires methods for quantitative synthesis of prior research, yet current meta-analysis methods limit aggregation to studies with similar designs. Here we describe and validate Generalized Model Aggregation (GMA), which allows researchers to combine prior estimated models of a phenomenon into a quantitative meta-model, while imposing few restrictions on the structure of prior models or on the meta-model. In an empirical validation, building on 27 published equations from 16 studies, GMA provides a predictive equation for Basal Metabolic Rate that outperforms existing models, identifies novel nonlinearities, and estimates biases in various measurement methods. Additional numerical examples demonstrate the ability of GMA to obtain unbiased estimates from potentially mis-specified prior studies. Thus, in various domains, GMA can leverage previous findings to compare alternative theories, advance new models, and assess the reliability of prior studies, extending meta-analysis toolbox to many new problems.

## Introduction

Normal science progresses when scientists build on prior research to extend, test, and apply theories of biological, physical, and social phenomena [1]. Aggregating the findings from the existing literature therefore plays a critical role in advancing the sciences. In most cases, qualitative review articles provide the method for taking stock of what is known, but offer little quantitative guidance for combining those results. Quantitative combination of prior models is, however, needed for prediction, model comparison, hypothesis testing, and cost-benefit analysis.

The current approach to quantitative aggregation of prior research uses various meta-analysis techniques [2]. The common approaches to meta-analysis combine findings from multiple studies each measuring the impact of one explanatory variable (e.g., a treatment) on one response variable (e.g., a health outcome). Therefore, they seek better estimates of one specific effect across multiple studies. Fixed effect meta-analyses assume the underlying effect is the same across different studies, while random effect models allow for a distribution (typically normal) for the underlying effect across studies and estimate the parameters of that distribution. Such meta-analyses are often used in biomedical research to aggregate multiple statistical estimates [3], e.g., from clinical trials, into more reliable estimates of causal effects [4]. More complex methods are being devised to enable meta-analysis where simple methods are not applicable. Multivariate meta-analysis methods combine prior studies that include multiple outcomes, e.g., disease-free and overall survival in cancer research [5]. These methods utilize the correlation among those outcomes and across studies to come up with tighter estimates for each underlying effect [6]. A related stream of research uses meta-regression to assess how the effect of interest is modified by factors that vary across prior studies [7]. Despite their utility, current meta-analysis methods can only combine relationships between explanatory and response variables that use the same functional forms and variable measures across the prior studies [8]. Moreover, more complex meta-analysis methods may rely on hard-to-verify assumptions such as multi-variate normality for correlated effects in multivariate meta-analysis and expose the study to risk of data-drudging (e.g., by considering different effect modifiers in meta-regression [9]). Therefore, reliable and transparent methods for quantitative aggregation of findings do not exist when prior studies use different statistical models, different subsets of potential explanatory variables, or different transformations on the variables they include.

Despite these limitations, the rapid growth of scientific literature has promoted increasing applications of meta-analysis. Publications listed in nine major databases (Web of Science Core Collection, MEDLINE, Biological Abstracts, Zoological Records, BIOSIS Citation Index, Data Citation Index, SciELO Citation Index, Current Contents Connect, and Derwent Innovations Index) with the term “meta-analysis” in the title show over 25-fold growth (from 1,247 in to 31,314) over the last decade, now reaching tens of thousands annually. Thus, the value of a broader method for quantitative aggregation of prior research can be immense across various disciplines. Consider a few examples.

- Over 125 studies in environmental science have analyzed the impact of the pesticide Atrazine on freshwater vertebrates, yet no quantitative conclusion can be drawn in the absence of a method to combine them [10].
- A meta-regression study combines 60 prior estimates of the impact of climate change on human violence [11], but its findings are questioned because the method does not account for cross-study correlations and mixes heterogeneous measures (e.g., linear, non-linear, and lagged effects) in the original studies [12].
- In energy research, multiple methods exist to estimate diffuse solar energy in a location using data from distant sensors [13], however, there is no method for proposing a model that aggregates these methods into a single estimating equation.
- In occupational health, at least 10 studies have estimated the effectiveness of workplace-based return-to-work interventions after injury or illness [14], yet the heterogeneity in study designs and statistical methods have precluded quantitative aggregation of these findings.
- In urban planning, a review found 45 published models of municipal solid waste generation [15]; given the various analytical methods applied, these studies have not been combined to provide a more general and reliable model.
- In obesity research, over 47 separate studies have estimated human basal metabolic rate (BMR) as a function of different body measures, such as fat mass (F), lean mass (L), body weight (BW), age, and height, among others [16]; combining these findings into a single equation would benefit research and practice.

In such settings, besides quantitative aggregation of prior findings, a general approach to aggregation could allow researchers to leverage the data previously collected by others to build, test, and compare alternative new theories, and assess the reliability of individual studies.

In this paper, we introduce the Generalized Model Aggregation (GMA) method. GMA uses available summary statistics from prior studies to estimate a meta-model that, when simulated, can replicate those original statistics. GMA provides consistent and reliable estimates, requires few restrictions on the structure of the meta-model or previous studies, can correct for biases in prior studies due to missing variables and model mis-specification, allows for both fixed effect and random effects models, and accommodates statistics for hypothesis testing and model selection. Moreover, the GMA approach relies on few assumptions about model structure and underlying effects, offering transparent and easy-to-understand results even in aggregating heterogeneous prior studies. Therefore, GMA enables quantitative model aggregation, theory building, and theory testing in a wide range of applications that previously relied only on qualitative literature reviews to synthesize existing findings. We demonstrate the GMA method in multiple simulated scenarios and an empirical validation.

## Methods

### Background and overview

**Fig 1:**
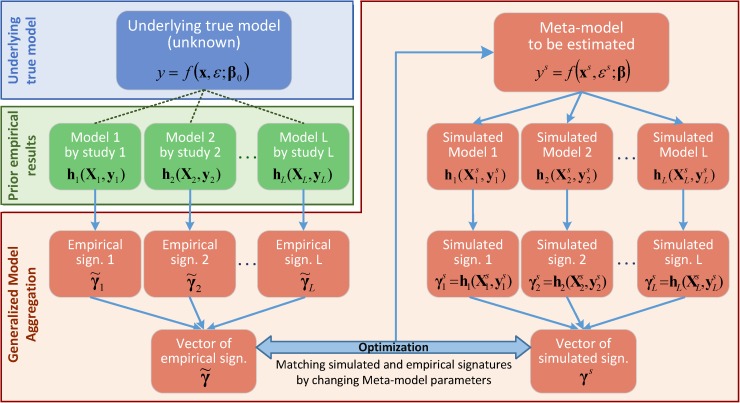
Overview of GMA. Prior studies provide the vector of empirical signatures, ${\tilde{\gamma }}_{l}$. The hypothesized meta-model is estimated by simulating those signatures and matching them against empirical ones.

The intuition behind GMA is simple and summarized in Fig 1. Prior studies provide statistical estimates (denoted as *signatures*, e.g., regression coefficients, correlation matrices, and variance of effect sizes across prior studies) that, even if biased and incomplete, include relevant information about the phenomenon of interest, i.e., the data generating process. A meta-model corresponds well to the real data generating process if the same statistical operations that generated the empirical signatures of prior studies lead to similar signatures when applied to simulated data from the meta-model. Thus, by matching the simulated signatures from a meta-model against the empirical signatures of prior studies, we can estimate the parameters of the meta-model. The resulting meta-model aggregates prior research by embedding into a single model the quantitative information from all prior studies and the variations across them.

The idea of matching simulated summary statistics against the empirical ones is well-established. For example, it is the basic idea of the method of simulated moments [17], and has been applied to estimating complex models with intractable likelihood functions using indirect inference [18, 19], estimating dynamic network models using the stochastic actor-based approach [20], approximate Bayesian computation for estimating posterior without calculating likelihood [21], as well as in model calibration in sample survey completion using external data [22]. In these various methods matching simulated statistics against empirical ones is the basic idea, but each method has a different goal, and thus various approaches for selection of summary statistics, definition of the models and linking functions, generation of simulations, and optimization are pursued. To our knowledge, GMA provides the first formal method to apply this basic idea to meta-analysis and complex model aggregation problems.

### Generalized model aggregation

Consider a data generating process *y* = *f*(**x**,*ε*;**β**_0_) where the response variable, *y*, is a (possibly nonlinear) function of explanatory variables **x** = [*x*_*i*_]; *i* = 1,2,…,*I* where **β**_0_ = [*β*_0,*j*_]; *j* = 1,2,…,*J* are the function’s parameters and *ε* is a random error term. Errors can have an arbitrary joint distribution, the parameters of which are included in **β**_0_, so that by estimating the parameter vector **β**_0_ the data generating model is fully specified. This general model can accommodate any explicit functional relationship between a group of variables, including dynamic models, as well as fixed and random effects models, among others.

In GMA, we estimate the data generating model by utilizing the results of *L* available studies that have been previously estimated based on data from the data generating process. Suppose that each study includes a subset of *I*_*l*_ ≤ *I*; *l* = 1,2,…,*L* explanatory variables and a response variable, denoted by $X_{l}={\left[\begin{array}{cccc} x_{l1}^{T} & x_{l2}^{T} & \cdots & x_{{ln}_{l}}^{T} \end{array}\right]}^{T}$ and *y*_*l*_ respectively, and each study estimates the parameters of a functional relationship between *y*_*l*_ and **x**_*l*_ using *n*_*l*_ observations. Let ${\tilde{\gamma }}_{l}$ be the empirical signatures from study *l*, which may include estimated model parameters, goodness of fit measures, correlation matrices, or any metric reported in the original study that captures some statistical regularity among **x**_*l*_ and **y**_*l*_. The set of possible signatures is limited to the statistics reported in prior studies but there is no simple rule for identifying the right signatures. Typically, good signatures are sensitive to changes in the parameters of the meta-model, providing a clear signal for model identification, and have low variance. Note that the function by which ${\tilde{\gamma }}_{l}$ is estimated, **h**_*l*_(**X**_*l*_,**y**_*l*_), is known from the original study.

At the heart of GMA is the idea that if we simulate the true function *f* to generate **y**^*s*^ values given **β** and consistently simulated **X**^*s*^ values, and transform **X**^*s*^ and **y**^*s*^ values using the function $\gamma_{l}^{s}(\beta )=h_{l}\left(X_{l}^{s},y_{l}^{s}(\beta )\right)$, the resulting vector of statistics, $\gamma_{l}^{s}(\beta )$, would be close to the corresponding empirical signatures, ${\tilde{\gamma }}_{l}$. Specifically, $E\left({\tilde{\gamma }}_{l}-\gamma_{l}^{s}(\beta )\right)$ would converge to zero as *n*_*l*_ grows large. Therefore, the simulated counterparts of this expectation, $e_{l}\left({\tilde{\gamma }}_{l},X_{l}^{s},y_{l}^{s}(\beta )\right)=\frac{1}{S}\sum_{s=1}^{S}\left({\tilde{\gamma }}_{l}-\gamma_{l}^{s}(\beta )\right)$, would be small if the function used for generating simulations closely resembles the true data generating process. The same idea applies to the separate set of empirical signatures, ${\tilde{\gamma }}_{0}$, that capture between-study characteristics, e.g., between-study variance in some comparable effect sizes. Defining $e={\left[\begin{array}{cccc} e_{0}^{T} & e_{1}^{T} & \cdots & e_{L}^{T} \end{array}\right]}^{T}$, where $e_{0}^{T}$ is the error in predicting between-study signatures and $e_{i}^{T}$ the difference between simulated and empirical signatures from study *i*, the parameters of the data generating model, **β**, are then estimated by minimizing a weighted squared zero function, $\underset{\beta }{\hat{\beta }=\arg\;\min}\{e^{T}We\}$, where **W** is a positive semi-definite weight matrix.

GMA estimates share their theoretical underpinnings with the method of simulated moments [17, 23] and indirect inference [18, 19] giving them many appealing statistical properties. They do not require explicit likelihood functions and are consistent under mild assumptions, specifically, as long as the underlying signatures are consistent and include enough information to identify the model (**Notes D** and **E in S1 File** include conditions and proofs). Estimated parameters are asymptotically multivariate normal under many common scenarios (see **Note F in S1 File**), providing a direct and simple path to confidence interval estimation and hypothesis testing. While any positive semi-definite weighting matrix can be used for estimation, **W** is optimal (in terms of minimizing the variance of estimated parameters; see **Note G in S1 File**) when it is proportional to the inverse of the covariance matrix of the empirical signatures. That covariance matrix is unknown, however it can be estimated iteratively from the estimated model (See below, and **Note G in S1 File** for details). More generally, the covariance matrix for **β**, around the true value, can be estimated as **DW**^−1^**D** (**Note H in S1 File**), where **D** is the partial derivative of signatures with respect to model parameters **β**; and general confidence intervals can be obtained using bootstrapping methods [24] (**Notes I** and **C.3 in S1 File**). Using the optimal weighting matrix (${\hat{W}}^{*}$), the goodness of fit for a meta-model can be assessed using the statistic $\chi_{0}=\frac{S}{1+S}\underset{\beta }{\min}{\left[\sqrt{n}\frac{1}{S}\sum_{s=1}^{S}(\tilde{\gamma }-\gamma_{}^{s}(\beta ))\right]}^{T}{\hat{W}}^{*}\left[\frac{1}{S}\sum_{s=1}^{S}(\tilde{\gamma }-\gamma_{}^{s}(\beta ))\right]$, which asymptotically follows a Chi-square distribution with $\text{dim}(\tilde{\gamma })-\text{dim}(\beta )$ degrees of freedom under the null hypothesis that the estimated function is the true data generating process (**Note J in S1 File**). The goodness of fit measure provides a test to identify the studies with signatures most divergent from other prior studies, and assists with diagnosis of the underlying causes of such discrepancy. Models with different numbers of parameters can be compared using a Model Selection Criterion (MSC) that balances goodness of fit against the number of estimated parameters [25] and the model with the smallest MSC value is preferred (**Note K in S1 File**).

Implementing GMA requires samples of (simulated) explanatory variables, **X**^*s*^, consistent with the empirical distributions, and an appropriate **W** to be used in the optimization that estimates **β**. Samples of explanatory variables can be obtained in several ways: (i) Existing empirical data sources may include such data. For example, in aggregating studies of the determinants of BMR, publicly available data from the NHANES includes the explanatory variables related to BMR estimation (but not the BMR itself.) (ii) Samples can be simulated based on information provided in prior studies, e.g., the reported mean and covariance matrix of **x**_*l*_. In this case correlation/covariance matrices of prior studies need to be combined using one of the existing methods (e.g., see [26]) to provide a single matrix for simulating samples. (iii) When prior studies do not report correlation or covariance matrices of explanatory variables, the joint distribution of **x** may be modeled with additional parameters. That auxiliary model can be estimated simultaneously or separately using the GMA to match any information we have about the distribution of explanatory variables (see **Notes B.2** and **B.3 in S1 File** for a demonstration and comparison across these methods). In fact, in some common settings the information in reported regression coefficients (signatures) allow one to estimate those distributions (see **Note B.2 in S1 File** for one such example). Finally, if available, original data from any of the prior studies can also be directly used instead of samples of **X**^*s*^.

An efficient estimate of **W** is typically not available at the outset. That matrix can be estimated iteratively, e.g., starting with a diagonal **W** matrix with elements proportional to the reciprocal of squared elements of $\tilde{\gamma }$, estimating **β** using this initial weight matrix, then simulating the signatures many times to estimate their covariance matrix, and using the inverse of this estimate as **W** in the next round of **β** estimation. Similar to the method of simulated moments [23], in most problems the process converges to an efficient estimate of **W** in just a few iterations.

**Fig 2:**
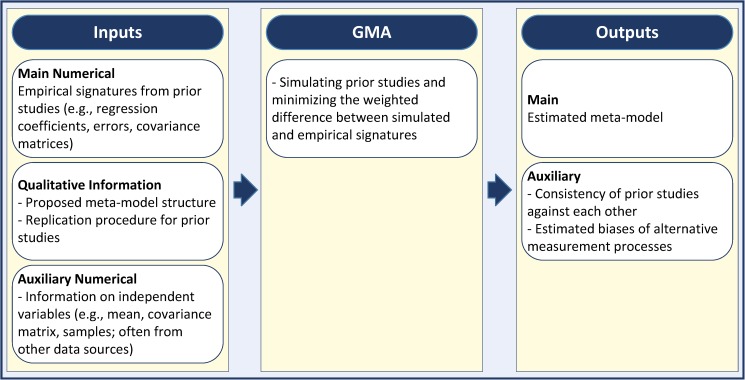
GMA and its inputs and outputs.

Fig 2 summarizes the inputs and outputs of GMA. In short, the inputs to GMA include empirical signatures from prior studies, the information needed to replicate each prior study, and data sources that allow us to create samples of independent variables. This information is then used by GMA to estimate the parameters of a data generating function (meta-model), the output of which can be matched against the empirical signatures. Besides estimating the meta-model, GMA also offers useful output information on the reliability of prior studies and their measurement methods. **S1 File** provides the details of the GMA algorithm, proof of consistency, distribution of parameter estimates and hypothesis testing procedure, optimal weight function (**W**), procedure for generating simulated inputs (**X**^*s*^) and goodness of fit measures. These and statistical proofs and additional information on various experimental settings are in **Notes A-K in S1 File**. We also provide the code (in MATLAB) and instructions required for replicating the analysis and new GMA applications (see **S2 File** and **S3 File**).

## Results

### Experimental results

To validate GMA, we first test it on simulated problems where the underlying data generating model is known. In each case, a true data generating process, *f*(**X**;**β**_0_), is specified, raw data are generated from the process and (imitations of) prior studies are estimated. Coefficients and error statistics from those studies are then utilized as signatures in GMA ($\tilde{\gamma }$) to estimate the parameters of the data generating model ($\hat{\beta }$). Finally, these estimates are compared to the true values (**β**_0_).

**Fig 3:**
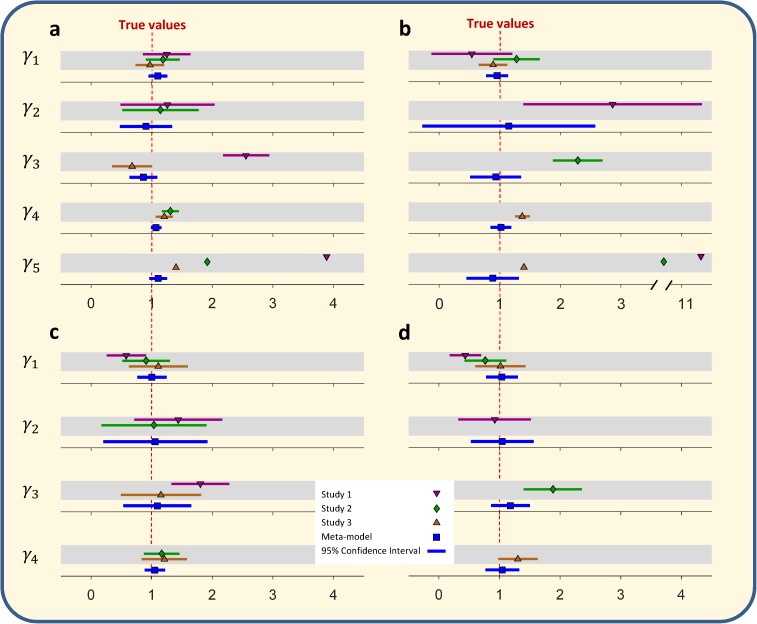
Aggregation of three “prior” study regressions across four scenarios. a) Estimated parameters of a linear generating process (meta-model) **y** = *β*_0_+*β*_1_**x**_1_+*β*_2_**x**_**2**_+*β*_3_**x**_3_+**ε**. Three prior studies of the form **y** = *β*_0_+*β*_*i*_**x**_*i*_+*β*_*j*_**x**_*j*_+**ε**; (*i*,*j* = {1,2,3}; *i* ≠ *j*) are estimated and their coefficients are reported within the gray bars. b) Similar to a, but prior studies estimate models of the form **y** = *β*_0_+*β*_*i*_**x**_*i*_+**ε**; (*i* = {1,2,3}). c) Similar to a, but using binary outcomes and logistic regression meta-model of the form Pr(**y** = 1) = (1+exp(−(*β*_0_+*β*_1_**x**_1_+*β*_2_**x**_**2**_+*β*_3_**x**_3_)))^−1^ with prior studies including only two of the three explanatory variables and a constant. d) Similar to c, but only including one explanatory variable and a constant in each prior study. In (a), (b), (c), and (d), *γ*_1_ represents the intercept and *γ*_2_, *γ*_3_, and *γ*_4_ represent the coefficients of **x**_1_, **x**_2_, and **x**_3_, respectively, both in “prior” study regressions and meta-model. In (a) and (b), *γ*_5_ represents MSE in “prior” study regressions and the estimated standard deviation of the error term in the meta-model.

Seven scenarios across five model structures are explored. In Scenario 1 (Fig 3A), the “true” data generating process is **y** = 1+**x**_1_+**x**_**2**_+**x**_**3**_+**ε**. Regression results from three “prior” studies, all assumed to be linear regressions, are considered. Each imitated prior study uses a constant and two of the three explanatory variables to estimate **y** (three coefficients estimated). The prior models are therefore mis-specified. Further, the explanatory variables are correlated and therefore the estimated coefficients of the prior studies are potentially different from the true coefficients. We apply GMA to estimate the true model, using those three coefficients and the mean squared error (MSE) of each study, a total of 4×3 = 12 signatures. We simulate the explanatory variables, (**X**^*s*^), based on mean vectors and covariance matrices reported in the three prior studies. Fig 3A reports results from one typical example. GMA estimates correctly infer the true data generating process, with rather tight confidence intervals.

GMA is similarly effective in three other linear models. In Scenario 2 (Fig 3B), the complexity of the problem is increased. The same true data generating process is used but prior studies now include a constant term and only one explanatory variable. Thus after including MSE each “prior” study offers three signatures for a total of 9 signatures, which are used to estimate the meta-model. Scenario 3 (Fig 3C) reports an experiment parallel to Scenario 1 (i.e., a constant and two of the three explanatory variables included in each prior study), but this time the outcome is binary following a logistic model as Pr(**y** = 1) = (1+exp(−(1+**x**_1_+**x**_**2**_+**x**_**3**_)))^−1^, and the three “prior” studies estimate a logistic regression. Scenario 4 (Fig 3D) extends the logistic regression example to a case where only one of the explanatory variables and a constant are included in each prior study regression.

In all four scenarios GMA extracts unbiased estimates of the true data generating process with 95% confidence intervals comparable to or tighter than those in the original studies. Even when prior studies are missing important variables and do not include the true effects in their confidence intervals, their signatures contain information, and GMA can extract that information to yield better estimates (**Note B.1 in S1 File**). In fact, in the above examples, GMA is effective even though *none* of the prior studies include all the relevant explanatory variables. Moreover, GMA may result in corrected effects that fall outside of the range of effects estimated by all prior studies (e.g., see estimated effect for γ_2_ and γ_4_ in Fig 3A), which is infeasible in common meta-analysis methods that use weighted averaging of prior estimates. Furthermore, GMA offers a method to resolve the apparent inconsistency across coefficients from the prior studies. For example in Scenario 1, “prior” study 1 estimates the (mis-specified) model *y* = *γ*_1_ + *γ*_2_*x*_1_ + γ_3_*x*_2_ + *ϵ*, finding *γ*_3_ = 2.55 with a 95% confidence interval of (2.2, 2.9), which excludes the true value (of 1), and study 2 estimates = *γ*_1_ + *γ*_3_*x*_2_ + γ_4_*x*_3_ + *ϵ*, finding *γ*_3_ = 0.7 with a 95% confidence interval of (0.4, 1.0). GMA uses the information in these mis-specified models to estimate *β*_2_ = 0.9, with a 95% confidence interval of (0.7, 1.1). GMA resolves the apparent inconsistency by showing how the estimates of the prior studies differ from each other and from the true value due to model mis-specification and the correlations among the explanatory variables. In the supporting information, we assess the impact of various methods for generating the explanatory variables and their potential errors (**Table B in S1 File**). Moreover, to check the robustness of these findings, the experiments are repeated 1,000 times for scenario one (**Table A in S1 File**), and the large sample results are consistent with the basic findings reported above.

In Scenario 5, we explore GMA’s ability to infer a continuous nonlinear data-generating process from prior analyses of variance (ANOVA) on categorical data. We use a true data generating process of the form **y** = 1+**x**_1_+**x**_**2**_+**x**_1_**x**_**2**_+**ε**∼N(0,1). Three “prior” ANOVA studies with sample sizes *n* = 100 are considered where factors (*x* variables) are categorized into three (for **x**_1_) and four (for **x**_2_) groups based on the 33^th^ and 66^th^ percentiles for **x**_1_ and the first, second, and third quartiles for **x**_2_. Two prior one-way ANOVA studies assess the treatment effect of **x**_1_ and **x**_2_ separately, and a third study looks at both factors, but includes no interaction term. In this example, the prior studies include data limited on multiple fronts: continuous variables are discretized, interaction effects are not considered, and variables are missing in two of the prior studies, therefore, it may be hard to identify the underlying model. To conduct GMA, main effects, Mean Sum of Squares due to Treatment (MST), and Mean Squared Errors (MSE) are included as signatures, creating 6, 7, and 11 signatures for the three studies, respectively. GMA is then used to estimate a data generating process of the form **y** = *β*_0_+*β*_1_**x**_1_+*β*_2_**x**_**2**_+*β*_3_**x**_1_**x**_**2**_+**ε**∼N(0,*β*_4_). Over 1,000 replications of this experiment, we are able to consistently identify unbiased estimates for the underlying data generating process as well as accurate analytical confidence intervals, even though the prior studies ignored the continuous nature of the data (used categorical explanatory variables) and included no interaction terms (see **Note B.4 in S1 File** for additional details).

**Fig 4:**
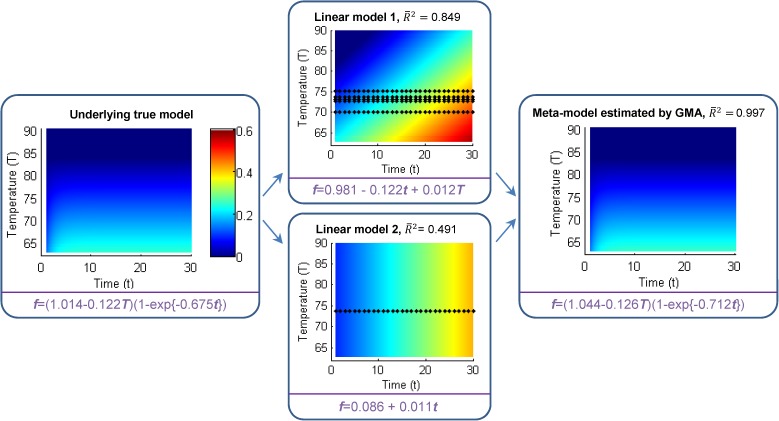
Comparison of two linear models and the nonlinear meta-model with the underlying true model. The predicted outcome is fluid leakage rate and its expected value under the true data generating process (left), and each model is shown using color maps. Black dots in the two middle charts identify the original data points used in estimation of the two linear models. However, these “raw” data points are not used in GMA estimation, only the coefficients of the two linear models (3+2 coefficients) and two R^2^ terms (total of 7 signatures) are used for estimation of the non-linear meta-model (graphed on the right).

Scenario 6 explores GMA’s applicability to nonlinear models. Specifically, following a prior study [27], the true data generating process for transmission fluid leakage in transmission systems is assumed to be a nonlinear function of time and temperature. Simulated prior studies include two linear models, one only including time, and the other including both time and temperature. The results of one experiment are reported in Fig 4 (with additional details in **Table E in S1 File**). Again, GMA is able to aggregate mis-specified prior studies and accurately estimate the true model. This example is noteworthy because the nonlinearities of the true model, not included in prior studies, are identified by GMA.

Finally, in the last scenario we assess GMA’s ability to estimate random effects models and compare it with the standard random effects method that is the workhorse of classical meta-analysis [28]. In this comparison, we use as signatures five “prior” effect sizes, along with their reported within-study variances, and using the GMA estimate the summary effect size and the between-study variance in the mean effects. We compare the GMA results with standard random effects meta-analysis that uses the DerSimonian and Laird method [29]. We vary the true between-study variance across five levels to assess the sensitivity of comparisons to the level of noise. In data generation, we follow the basic assumptions of standard meta-analysis, i.e., normally distributed within and between study error terms. Since not required by GMA, these assumptions favor the analytical method used in classical meta-analysis and thus provide a conservative test, nevertheless, across 200 replications for each of the five parameter set-ups, GMA does as well as, or statistically better than, the classical method in terms of estimation errors. Details of this scenario are discussed in **Note B.6 in S1 File**.

In the previous scenarios, we assumed no measurement errors for explanatory variables. However, measurement errors are common and can lead to biased parameter estimates in the prior studies, and thus in GMA results. Therefore, it is important to account for those errors when they are expected to be large. There are multiple methods in the literature for correcting for measurement error in traditional meta-analysis [30, 31] and it may be possible to adapt some of those to GMA. Specifically, it is easy to incorporate a measurement error into simulations of meta-model in GMA, by adding a (simulated) error term to the simulated explanatory variables, before replicating the prior studies on this simulated, measured, data. If good estimates for the magnitude of those measurement errors are available, those can be used in the simulations, and the estimation of the resulting model can progress without any additional parameters. More challenging is the case when priors for measurement error size are not available. In this case the variances of measurement errors could be added to the unknown parameters to be estimated by GMA, and the augmented model estimated as before. We explored this idea in the case of scenario one, assuming normally distributed measurement noise levels as a fraction (δ between 0 and 1) of the underlying variables’ actual standard deviations, and estimating the extended meta-model with the resulting three additional unknown parameters. Repeating this analysis 100 times for each value of δ, we found that measurement error increases the variance in parameter estimates and the width of the confidence intervals. Nevertheless, these confidence intervals are still largely reliable, and GMA offers estimates for measurement noise that were otherwise not available. The details for this analysis are reported in **Note B.7 in S1 File**.

### Empirical example

Basal metabolic rate (BMR) is the largest component of human energy expenditure and accurate estimates are critical for understanding human metabolism, developing obesity and malnutrition interventions, and identifying patients with metabolic abnormalities, among others. As an empirical validation of GMA, we aggregate prior studies of the determinants of BMR and compare the predictive power of the resulting meta-model with existing models in the literature. We focus on a single population group, white males over 18 years of age. A recent review of the literature [12] yields 16 studies reporting 27 regression models that estimated equations for BMR (in Kcal/Day) for members of this population and included sufficient details for simulated replication. Those regressions use different samples of the population and include various subsets of height (H; in cm), weight (W; in Kg), fat mass (F; in Kg), lean mass (L; in Kg) and age (A; in Years) as explanatory variables. The prior studies do not report the covariance or correlation matrices of their samples. However, data on these explanatory variables are available in many public databases, including the U.S. National Health and Nutrition Examination Survey (NHANES) [32]. We thus sample these explanatory variables from NHANES. We use sampling functions that are separately estimated by GMA, replicating, as signatures, the reported mean and variances of explanatory variables for each prior study (see **Note C.1 in S1 File** for details on generation of explanatory variables). In replicating prior studies, we include additional parameters to capture variations due to different technologies used to measure BMR and fat mass. We then combine NHANES data, sampling functions, and signatures from prior studies to generate the GMA estimates for four alternative meta-model specifications. These alternatives include both linear and nonlinear candidates derived from detailed modeling of human metabolism and weight dynamics [33]. Overall results are summarized in Table 1. The resulting best fitting equation based on MSC is:

BMR=-3526+3.6H+11F-5.8L-2.6A-130.4ln(F)+1299.3ln(L)+N(0,136)

**Table 1:** Estimates for alternative BMR meta-model specifications.

Alternative Meta-model Estimates	MSC^a^
BMR = 558 + 2.8H + 7.5F + 12L - 3.1A + N(0,170)	2,676
BMR = 851 + 1.1H + 8.7F + 13L - 3A - 3.3BMI^b^ + N(0,172)	2,722
BMR = 231 + 4.4H + 3.1F + 16.2L - 2.4A + 0.06F^2^ - 0.03L^2^ + N(0,128)	2,429
BMR = -3526 + 3.6H + 11F - 5.8L - 2.6A - 130.4 ln(F) + 1299.3 ln(L) + N(0,136)	**2,390**

^a^Model Selection Criterion, MSC = χ_0_ + 2 dim(β).

^b^Body Mass Index (BMI), a common measure of obesity, is weight divided by height squared.

The accuracy of this model is compared against alternatives in predicting BMR in an empirical validation sample of 159 male subjects [34] not used in the estimation or prior studies. Our model’s mean absolute percentage error (MAPE) of 6.66% is lower than any of the 27 equations used in creating the meta-model, which have MAPEs in the range 7.17%-14.36%. Our model also proves more accurate than the equations by World Health Organization [35] (MAPE = 7.98%) and Institute of Medicine [36] (MAPE = 7.66%), which are widely adopted by researchers and practitioners and are considered among the most accurate models. The improvements in accuracy are substantial when we consider the lower bound on error due to unobservable individual variations. For example, if the same model structure as our best-fitting equation from GMA was fitted to the validation sample, thus providing the lowest error for this sample, its MAPE would be 6.60% (**Note C.6 in S1 File**).

While the validation dataset is not large for the standards of many typical statistical analyses, it is noteworthy in this context. First, the sample is a relatively large one in this literature given the costs and complexities involved in good measurements of BMR and Fat Mass. Indeed, we found only 3 prior studies that included larger samples, while the vast majority of prior research had used much smaller samples (typically under 50 subjects). Moreover, we needed a validation sample that was not used in prior published BMR estimation, so that we would not unintentionally bias our own model selection procedure, or contaminate the input data into GMA when using prior studies that had utilized the validation sample. The validation sample we used offered a satisfying resolution to both concerns.

Besides providing more accurate equations without using any individual level data, GMA provides three additional insights. First, it identifies a statistically significant nonlinearity in the change in BMR as a function of *L* and *F*. None of the prior studies included a nonlinear term, yet our results suggest a nonlinearity can be inferred and improves prediction, a finding which is consistent with detailed (organ level) modeling of BMR that finds different organs respond differently to changes in body weight [37]. Second, in the process of estimating the meta-model we included parameters that represent the biases in different methods for the measurement of BMR and fat mass. Therefore, this application of GMA also provides estimates for how different measurement methods compare with each other, which can be used to calibrate different measurement methods (**Note C.4 in S1 File**). Finally, using the changes in goodness of fit measure induced by exclusion of each prior study, we assess the consistency of previous research and identify the outliers (**Note C.5** and **Table H in S1 File**). We also provide a comparison of the analytical confidence intervals with those obtained by bootstrapping for this empirical example, finding that the two methods are fairly consistent (**Table J in S1 File**).

## Discussion

GMA provides a flexible method to quantitatively combine diverse statistical findings. GMA extends meta-analysis to more heterogeneous sets of underlying studies that vary in design and variable operationalization. At the heart of this method is the idea that any statistic reported in prior studies has information about, i.e., a signature of, the data generating process. The development of GMA, a method to piece together many such signatures, further highlights the importance of detailed reporting and replicability of scientific studies. For example, there is much useful information in the covariance matrix of explanatory variables and reporting that, even if in an online appendix, would be very valuable for other researchers. GMA can facilitate the iterations of theory building and theory testing central to scientific method. By allowing researchers to formally estimate and compare new models against prior results, GMA provides faster feedback about the usefulness of new models and theories. Moreover, GMA can be used to assess the consistency of new detailed models of a phenomenon against more aggregate empirical findings. For example, detailed models of BMR defined at the body-organ level could be constructed and partially estimated by GMA against prior BMR equations. While domain-specific research informs many disaggregated parameters (e.g., the metabolic rates of brain and liver), GMA could ensure the aggregation of those components into a model that is consistent with overall findings (e.g., BMR change over age and due to weight changes). Such applications promise a more fruitful dialogue between mechanism-based and statistical models. GMA can also help resolve apparent inconsistencies among prior studies. For example, the effect of different measurement methods for similar concepts could be estimated and inconsistencies due to measurement separated from those due to missing variables or heterogeneity in the underlying samples.

This paper introduces the idea of GMA and motivates future research to apply, elaborate, and expand the method. First, to keep the method general, we used a simulation-based approach that can use any signature with a squared error matching function. More efficient likelihood based linking functions could be devised for well-behaved subsets of empirical signatures. Second, we offered various methods for generating samples of explanatory variables. Given the importance of these samples as inputs to GMA, future research should seek additional methods, assess the pros and cons of those methods, and investigate the sensitivity of results to various degrees of inconsistency between these samples and those from the data generating process. Third, we showed that GMA does a fine job in estimating traditional random effects meta-analysis models. Systematic comparisons with other meta-analysis methods is another promising area of research. Forth, a more detailed treatment of measurement error and conditions under which GMA can estimate that is a promising area for future studies. Moreover, theoretical research can focus on the properties of effective signatures and the minimum set of signatures required for identifying a given model. Finally, the extent of usefulness of GMA will only be known when more empirical applications are conducted. We hope this paper motivates many such applications.

GMA may overcome some of the common challenges faced by meta-analysis by accommodating broad model specifications, including study-specific effects, and potential omitted variable biases [38, 39]. Nevertheless, GMA is no panacea and its use will be limited to settings where it is empirically and computationally feasible and its fundamental assumptions are valid. Data required for simulating consistent samples of explanatory variables may not be available from prior studies or other data sources. Computational costs of GMA scale with the costs of simulating the prior studies and the number of simulations needed in the optimization (estimation) step. These costs are often limited when prior studies only include analytical or convex estimation problems: the most demanding of our analyses, the BMR example, required about 45 minutes of computation on a standard laptop. However, those costs may become prohibitive if replicating each prior study is computationally expensive or the optimization payoff landscape is very complex. More conceptually, GMA assumes that the different prior studies tackle the same underlying phenomenon, that those studies are statistically representative of the population of interest, and that the prior studies provide enough details to enable their replication. These assumptions may be violated [40]. If prior studies come from different phenomena (e.g., BMR equations for mice and human subjects) the imposition of a single meta-model would be unrealistic [41, 42]. Publication [43] and design biases [44] may lead to uneven pools of existing studies, which, if not explicitly modeled, can bias GMA outcomes. Moreover, poor replicability plagues many research reports [45, 46] complicating the simulated replication of the original studies needed by GMA. As with any meta-analysis method, careless use of GMA can induce unjustified certainty by providing quantitative meta-models where the underlying studies should not be combined, are systematically biased, or are not replicable [47]. GMA’s goodness of fit measure allows for the identification of some of the heterogeneity problems in prior studies. Overall, our results suggest that careful application of GMA can provide new opportunities to learn from prior research, leverage existing data, build new theories, and test competing models.

## Supporting information

S1 File Supplementary Infromation Document. This document includes three main sections: (1) Supplementary Notes A-K, (2) Supplementary Tables A-L, and (3) Supplementary Figs A-B.
(PDF)

S2 File README for GMA Codes. This document presents: (1) instructions on how to read and execute the codes, (2) descriptions about the notations and functions in the codes, and (3) details of the codes.
(PDF)

S3 File GMA Codes. This zipped file includes GMA codes in ‘.m’ files. The codes are developed using MATLAB.
(ZIP)
