# Supplementary material for: A flexible method for aggregation of prior statistical findings
Source: PLoS One. 2017 Apr 6;12(4):e0175111. doi: 10.1371/journal.pone.0175111 (PMC5383132; doi:10.1371/journal.pone.0175111)
Supplement: S1 File — This document includes three main sections: (1) Supplementary Notes A-K, (2) Supplementary Tables A-L, and (3) Supplementary Figs A-B. (PDF) [file pone.0175111.s001.pdf]

Supplementary Information for

A Flexible Method for Aggregation of Prior Statistical Findings

Hazhir Rahmandad[1*], Mohammad S. Jalali[1], Kamran Paynabar[2]

[1] Sloan School of Management, Massachusetts Institute of Technology, Cambridge, Massachusetts, United States of America
[2] Industrial and Systems Engineering, Georgia Institute of Technology, Atlanta, Georgia, United States of America

***** Corresponding author
E-mail: hazhir@mit.edu (HR)

This document includes:

Supplementary Notes A-K
Supplementary Tables A-L
Supplementary Figures A-B

Supplementary Information

Table of Contents

Supplementary Notes ... 3
Note A- Recipe for GMA ... 3
Note B- Details of simulated scenarios ... 5
B.1-Scenarios 1-4 (linear models) ... 5
B.2-(Co)Estimating the correlation matrix of explanatory variables ... 5
B.3-Sensitivity of results to distribution of explanatory variables ... 5
B.4-Scenario 5 (ANOVA) ... 6
B.5-Scenario 6 (non-linear model) ... 6
B.6-Scenario 7 (random effects meta-analysis) ... 6
B.7-Impact of measurement noise ... 7
Note C- BMR estimation details ... 8
C.1-Samples of explanatory variables ... 8
C.2-Numerical stability and overconfidence in measures of fit ... 9
C.3-Bootstrapped confidence intervals ... 9
C.4-Variations in measurement methods ... 10
C.5-Goodness of fit and its determinants ... 10
C.6-Minimum theoretical error ... 11
Note D- Statistical setup and assumptions of GMA ... 13
Note E- Consistency of GMA estimators ... 14
Note F- Asymptotic distribution of estimators ... 15
Note G- Optimal choice of signatures' covariance matrix and its estimate ... 17
Note H- Asymptotic variance of meta-model estimate using delta method ... 18
Note I- Confidence intervals using bootstrapping methods ... 19
Note J- Goodness of fit test ... 19
Note K- Model selection criterion ... 20
Supplementary Tables ... 21
Supplementary Figures ... 35

Supplementary Notes

Note A- Recipe for GMA

Below we provide a recipe of the main steps to use GMA. The **Supplementary README** (**S2 File**) provides further details about the code files listed in each step as well as all other code files, including their actions, inputs, and outputs.

Step 1: Choose signatures and replicate prior models

Code file: *User_model_(l).m*

The first step is to identify empirical signatures reported in each prior study. For example, if prior study 1 reports a regression model, one can select the empirical signatures as the intercept of the model, variable coefficients, and a measure of the model's goodness of fit. Other prior studies might have different model structures and empirical signatures. In general, any statistic that provides relevant information about the phenomena of interest can be selected as a signature.

Once the signatures are extracted and entered into *User_model_(l).m* code files, the prior models should be replicated (in the same code files). This replication requires simulated data for explanatory and response variables—discussed in Step 2 below—and with that data, it samples from the explanatory variables and estimates the model outputs. The sample from the explanatory variables is drawn considering the study sample size, subject characteristics, and the variables used in the respective study. The same model structures reported in the prior studies should be replicated and their outputs are then chosen as simulated signatures. Note that the simulated signatures and their orders should be the same as their empirical counterparts.

Step 2: Generate simulated data for explanatory and response variables

Code files: *User_DataGeneration.m* and *User_SIGMA.m* for the explanatory variables
User_Meta_model.m for the response variable

To replicate prior models and estimate their simulated signatures, data for explanatory and response variables are needed. The multivariate explanatory variables (generated in *DataGeneration.m*) should include all variables used in the prior models. A hypothesized meta-model (specified in *Meta_model.m*) also produces the response variable. See the paper for more information about providing/simulating explanatory variables and developing a meta-model.

Step 3: Initiate the GMA and optimization solver

Code files: *User_GeneralInputs.m*
User_OptInitiation.m

This step is to identify parameters needed for GMA (e.g., the number of replications in equation $\mathbf{e}_l\left(\tilde{\boldsymbol{\gamma}}_l, \mathbf{X}_l^s, \mathbf{y}_l^s(\boldsymbol{\beta})\right)=\frac{1}{S}\sum_{s=1}^{S}\left(\tilde{\boldsymbol{\gamma}}_l-\boldsymbol{\gamma}_l^s(\boldsymbol{\beta})\right)$) and initial conditions for the optimization solver (e.g., initial value for $\boldsymbol{\beta}$ to start the optimization search). See the **Supplementary README (S2 File)** for more information.

Step 4: Weighting matrix, optimization and iteration

Code files: GMA_W_star.m GMA_Optimization.m GMA_ObjFn.m

The weighing matrix $\mathbf{W}$, a positive semi-definite matrix, can be estimated optimally when it is proportional to the inverse of the covariance matrix of the simulated signatures. However, simulated signatures are computed based on estimated $\boldsymbol{\beta}$ while no estimated $\boldsymbol{\beta}$ is available at the beginning. Hence, a two-step procedure is used. In the first step, the we use a diagonal $\mathbf{W}$ with diagonal elements inverse of square of empirical signatures (and the non-diagonal elements be zero). Once the meta-model is estimated using this initial $\mathbf{W}$, the estimated $\boldsymbol{\beta}$ can be replaced for a second step using a more efficient $\mathbf{W}$ which is the inverse of the covariance matrix of simulated signatures.

Two implementation considerations need attention. First, to estimate the covariance matrix of simulated signatures, the number of replications to generate S series of simulated signatures should be large. This large number is only used once to better estimate the covariance matrix of simulated signatures, so it does not significantly increase the computational time. Second, if the estimated $\boldsymbol{\beta}$ in the second round is very different than the estimated $\boldsymbol{\beta}$ in the first round, it is recommended to continue with additional iterations until the estimated $\boldsymbol{\beta}$ converges (this number of iterations is presented by 'Opt_n' in the codes). Using $\mathbf{W}$ and optimization initial conditions identified in the previous step, optimization solver attempts to minimize a weighted squared of the difference between the vectors of simulated and empirical signatures. See the **Supplementary README (S2 File)** for more information about the codes.

Note B- Details of simulated scenarios

Below we report additional details on the simulated scenarios discussed in the paper.

B.1-Scenarios 1-4 (linear models)

In **Table A**, we provide details of 1000 replications of scenario 1. In each case, we report for the three prior studies and the meta-model, the mean, standard deviation, means of lower and upper 95% confidence interval bounds, and the percentage of 95% confidence intervals that incorporates the true parameter value.

In the first four scenarios, the explanatory variables for true data are normally distributed based on the mean and covariance matrix in **Table C**. In scenarios 1 and 3, GMA estimates are found by using mean and covariance values obtained from the (imitated) prior study samples. In scenarios 2 and 4, the covariance matrix of explanatory variables is assumed to be available from an auxiliary data source, which enables simulation of required samples for GMA. This assumption is similar to the approach used in the empirical example where samples of explanatory variables could be found from the NHANES data.

B.2-(Co)Estimating the correlation matrix of explanatory variables

In another comparison, reported in **Table B**, we assess GMA's ability to also estimate the correlation matrix of explanatory variables from the reported prior study coefficients. This case would be applicable when prior studies do not report the correlation/covariance matrices, and no other data source exists to inform the data generation of explanatory parameters. In this setting, we could use GMA to simultaneously estimate the parameters of joint distribution for the explanatory variables, as well as the data generation process for the response variable. Specifically, we assume prior studies have provided mean and variances of each explanatory variable but correlations should be estimated as part of GMA. In scenario 1, parameter estimates from prior studies (our signatures) include information about those correlations and thus the correlations could be estimated. The comparison with the case where correlations from prior studies are used for generating X variables is reported in **Table B**. Interestingly, the two cases perform rather similarly, with somewhat larger confidence intervals for model parameters when correlation matrix is also estimated using the GMA.

B.3-Sensitivity of results to distribution of explanatory variables

In **Figures A** and **B**, we assess the sensitivity of GMA to availability of accurate information on covariance matrix in scenarios 1 and 2. Specifically, we changed the precision of correlation matrix of explanatory variables using the following formula and assessed the quality of estimated meta-models:

$$\widetilde{Cr}_i = (1 - \varphi).\, Cr_i + \varphi.\, \text{Uniform}[-1,1]$$

Here $\widetilde{Cr}_i$ is one of the 3 elements of correlation matrix for explanatory variables used in generation of simulated data in GMA, Cr_i is the true value of the corresponding correlation element (see **Table C** for those values), and φ is a weight that allows us to assess a continuum between correct correlation matrix ($\varphi = 0$) and a fully random one ($\varphi = 1$). In simulations, we only use valid (positive semi-definite) correlation matrices that are generated from this formulation. The sum of squared errors in estimated parameters, weighted by corresponding variance of each parameter, follows an approximate Chi-squared distribution, so we report this error term and the fraction of 100 simulations for each φ that falls under the 95% confidence level (i.e., suggesting a good overall fit). As expected the precision of GMA depends on having relatively accurate estimates for the correlation matrix of explanatory variables. Moreover, the results are more sensitive in scenario 2, where a single explanatory variable is available from each prior study and errors in correlation matrix would not be compensated for by information in other signatures.

B.4-Scenario 5 (ANOVA)

Next, we report on scenario 5, the ANOVA study. Here sample data for prior studies are generated using the true model of $\mathbf{y} = 1 + \mathbf{x}_1 + \mathbf{x}_2 + \mathbf{x}_1\mathbf{x}_2 + \mathrm{N}(0,1)$, where $X_1 : \mathrm{N}(0,0.2)$, and $X_2 : \mathrm{N}(0,1)$, with a correlation of 0.4 between the two variables. Each prior study first categorizes the samples into three (for X_1) or four (for X_2) groups representing the relevant percentiles (e.g., different quartiles for X_2) for the corresponding factor. Then ANOVA is conducted on the sample assessing the existence of a main effect in each case, and main effects (category means-grand mean), MST, MSE, and grand mean are used as signatures in estimating a meta model of the form $\mathbf{y} = \beta_0 + \beta_1\mathbf{x}_1 + \beta_2\mathbf{x}_2 + \beta_3\mathbf{x}_1\mathbf{x}_2 + \mathrm{N}(0, \beta_4)$. **Table D** reports sample results from one instance of this experiment, as well as fraction of estimated confidence intervals in 1,000 iterations (at 80% and 95% confidence levels) which include the true parameter values.

B.5-Scenario 6 (non-linear model)

In **Table E**, we provide additional details on scenario 6, where two linear models are estimated on data from a nonlinear data generating process, and the correctly specified model structure is estimated for the meta-model. The true model is specified based on an empirical estimate which used a nonlinear regression and raw data to estimate the relationship. GMA is able to extract a precise estimator (coefficient of determination (R^2)=0.997) of the true model from rather mediocre linear approximations, with no access to any raw data.

B.6-Scenario 7 (random effects meta-analysis)

In scenario 7, we compare random effects meta-analysis using the GMA with classical methods. In this setup, the meta-analysis starts with prior reports on effect sizes and their standard deviation (within study standard deviation) and the goal is to combine those effect sizes, assuming that each effect size comes from a somewhat different data generating processes. While different, the true

effect sizes are all assumed to come from an underlying normal distribution with mean μ and standard variation σ. So the goal of meta-analysis is to estimate these two quantities based on the reported effect-size and within-study variance.

In simulating data from this process, we use a mean of μ=10 for true effect sizes in prior studies, and five levels of between study variance (σ^2={1, 2, 4, 8, 16}) to create five distinct scenarios. The observed effect size for each study is different from the true effect size (for that study) due to within study variation. The normally distributed within study error for each prior effect size has a mean zero and a variance that is randomly drawn from a Uniform [0-10] distribution. We repeat the analysis 200 times for each between study variance scenario. In each replication, five "prior" true effect sizes are generated using μ and σ, along with the corresponding within study variance for each prior study, ϵ_i. The "observed" effect sizes, μ_i, are then generated based on these within study variances and the true effect size that is not observed. Next, the μ_i and ϵ_i are supplied to both GMA and classical random-effects meta-analysis that uses the DerSimonian and Laird method [29], and estimates for μ and σ are obtained using each method. Neither method requires any other inputs. **Table F** reports the mean absolute error for estimates of μ and mean absolute percent error for estimates of σ using these two methods. For small values of σ (1, 2, and 4) GMA provides better estimates than classical meta-analysis. Estimates for μ are not significantly different across the two methods. GMA can accommodate other error distributions or more complex model setups but to keep the comparisons conservative we restrict this scenario to what can be done using classical meta-analysis tools.

B.7-Impact of measurement noise

To assess GMA's ability to identify measurement noise in explanatory variables and correct for that, we conducted 11 sets of analysis, each using the model specification in scenario one, with different levels of measurement noise. Specifically, we added a normally distributed measurement noise and changed the standard deviation of the measurement noise as a fraction, δ, of the standard deviation in each explanatory variable, changing δ between 0 and 1 with increments of 0.1. In each setting, we conducted 100 replications to assess large scale results. In each replication, we generated three prior studies with poorly measured explanatory variables, which in turn added to biases in the signatures. As signatures, we used the regression coefficients, error terms, and reported variances of the explanatory variables. We also added three measurement error standard deviation parameters (β_5 to β_7 in **Table G**) to the meta-model parameters (β_0 to β_4) and the true covariance matrix elements (not reported) to be estimated by GMA. **Table G** reports the results across 100 replications in each setting, including the 95% confidence interval (calculated using bootstrapping), the percentage of true parameters falling into this range, and the mean and median of the estimated parameters. Overall, the addition of these new parameters reduces the reliability of GMA estimates: confidence intervals are larger and parameter estimates less accurate. Nevertheless, GMA estimates largely fall within confidence intervals and estimates for measurement noise correctly increase with the increases in the actual measurement noise.

Note C- BMR estimation details

Table H provides additional details on the prior studies used for the BMR estimation case, including both estimated models and sample statistics for the explanatory variables. Parameter estimates for each model (i.e., the model intercept, variable coefficients, and standard deviation of the error term) and the coefficient of determination (R^2) are used as signatures, leading to a total of 115 signatures going into the GMA procedure.

Table I details the estimated meta-models and the uncertainty in their parameters and **Table L** reports the estimated effects of different measurement methods for BMR and FM.

C.1-Samples of explanatory variables

Samples of explanatory variables are needed in the BMR estimation case. While complexity of measuring BMR significantly reduces the availability of datasets useful for direct estimation of BMR, the explanatory variables going into BMR equation are easier to measure, and available in many datasets. In fact, a few waves of the NHANES dataset includes all the relevant variables for large samples of population. However, the samples used in prior studies of BMR are different from those of the NHANES sample, therefore we need to fine tune the sampling procedure from NHANES subjects to get study-specific samples that are closer in their distribution of explanatory variables to the original studies. The sampling process is conducted following two steps:

1) *Providing a large dataset of explanatory variables*

Despite the relatively large size of the NHANES database (in tens of thousands), the number of individual data in some age and BMI groups is not large enough, particularly for individuals at the tails of distributions, to accommodate many independent samples. For instance, there are few elderly subjects with BMI of larger than 40 (morbidly or super obese) in the NHANES, while one of the prior studies (Lazzer et al. [48]) needs 2,000 of those individuals—see **Table H**. This requires us to sample a large set of explanatory variables (*H*, *A*, *L*, and *F*) from a subpopulation not found in large numbers in the NHANES data.

Thus, rather than directly sampling from NHANES, we opt for simulating a large dataset based on joint distributions of explanatory variables in NHANES, from which we can then sample without any restrictions. Specifically, we first find transformations that turn the marginal distributions of the explanatory variables into normal distribution, then calculate the mean and covariance matrix of these transformed variables, assuming multivariate normality. We can then draw unlimited samples from this multivariate normal distribution and transform it back into corresponding explanatory variable sets. In the first step we estimated the following transformation functions to calculate multi-variate normal distributions from NHANES adult data: $H^{1.369}$, $L^{0.231}/H^{-0.251}$, and $F^{0.038}/H^{-0.981}$. For each various age groups (18-19, 20-25, 26-30,…, 80-85 years old) we estimate the mean and covariance matrix of these transformed statistics from NHANES data separately so that we account of impact of aging on the explanatory variables.

To sum up, this process allows for generating a larger sample of simulated individuals with statistics consistent with empirical NHANES samples. Compared using the relevant statistical test [49], the covariance matrices of this large sample of

simulated individuals and NHANES data (3,322 individuals from 2000-2008 rounds) are not statistically different.

2) *Draw sample data for each prior study*
Now that a large simulated dataset is available, samples can be drawn that are consistent with each prior study. To ensure this consistency (i.e., the drawn samples follow the same subject statistics as those presented in **Table H**), we use sampling functions. The sampling functions specify the probability that a simulated subject is included in the study, and we use the functional form:
$P(accepting\ a\ data\ point) = (1 + \exp(-(\sum_1^N w_n |m_n - u_n|)))^{-1}$ where w and m are parameters to be estimated for each prior study, and u is sample means for $W, H, A,$ $L,$ and F provided in **Table H**. The sampling function parameters (w and m) are separately estimated for each study to match the mean and variance of relevant sample statistics reported in each prior study (this can be seen as a simulated method of moments estimation). **Table K** reports these estimated sampling functions. With these functions in hand, the explanatory variables needed for GMA are generated by drawing from the large pool of data consistent with NHANES and accepted based on the study-specific sampling functions.

C.2-Numerical stability and overconfidence in measures of fit

Two implementation considerations need attention. First, given the large number of signatures, numerical stability in optimization steps could be an issue in inverting the empirical signatures' covariance matrix—for calculating the efficient weighting matrix. To address this issue, before inversion, we add a small positive number (epsilon) to the diagonal of the covariance matrix. This "ridging" of simulated covariance matrix is a common practice in many estimation settings where singular matrices may result from simulation.

Second, we suspect many researchers pick explanatory variables to get the best fit for the sample at hand. This customization of the regression function can lead to inflated fit measures that mislead GMA. For example, Piers et al. [50] provides the R^2 of 0.9 for young and elderly subjects using a small sample size (less than 40) and, with only one explanatory variable (W). While more complex models (i.e., those including more explanatory variables) with larger sample sizes report lower R^2—see **Table H**. If we put too much emphasis on matching this high R^2, GMA may unrealistically adjust other model coefficients. As a first step to fix such problems we inflate the variance in R^2 signatures by 0.01 to account for the additional variance that is due to sample-specific model customization and not sampling alone. This is a crude fix for the complex inter-study heterogeneity and more reliable solutions are a promising avenue for future research.

C.3-Bootstrapped confidence intervals

Table J reports results of estimating the 95% confidence intervals using bootstrap method (**Note I**) based on 200 iterations, for the best fitting model structure. These results are consistent with the confidence intervals using asymptotic derivation and are slightly larger and asymmetric, which is expected in comparing bootstrapped and analytical confidence intervals that rely on asymptotic normality.

C.4-Variations in measurement methods

Table L reports the impact of different measurement technologies on measured BMR and fat mass. Specifically, taking Deltatrac (indirect calorimetry, open circuit) and DXA (Dual-energy X-ray absorptiometry) as the standard methods for measuring BMR and Fat Mass, respectively, we define α parameters that specify the bias in other measurement methods compared to the benchmarks above. These bias parameters are incorporated into simulation models of prior studies to transform benchmark measure of BMR and FM to study-specific measures, which are then used in estimation. For example, if one study measures Fat Mass using Bioelectrical Impedance Analysis (BIA) with α=1.036, in creating its simulated signatures, Fat Mass values from the meta-model are multiplied by 1.036 to get the study-specific Fat Mass values entering the relevant regression. The value of 1.036 informs us that BIA relatively overestimates Fat Mass (this estimate is consistent with findings in Hendel, Gotfredsen (51)) and a similar interpretation can be applied to other bias estimates. Estimated bias parameters remain around 1, which is reassuring; when they are (statistically) significantly different from one, that indicates a notable difference between that measurement method and the benchmark. Therefore, we hope these estimates can prove helpful for understanding the differences among prior studies and offering a quantitative method for comparing and calibrating alternative measurement methods.

C.5-Goodness of fit and its determinants

In our BMR example, the goodness of fit measure is calculated at 2,356 which rejects the hypothesis that the estimated meta-model is fully consistent with the empirical signatures (the χ^2 test statistic at 95% confidence level is 122.1). Given the large number of signatures (115 in this example), this result should not be surprising. Two potential explanations can be used to understand these results. First, inconsistencies among the empirical signatures may be induced by various biases in prior studies (e.g., publication bias may lead to the presentation of only a subset of statistical results with some unobservable bias), or by use of populations different from those we simulate (e.g., due to limited information reported on the explanatory variables). Second, we only explored a handful of alternative models; better model structures may be hypothesized that could reconcile the various empirical signatures.

We further explore the variations among the prior studies by calculating the changes in goodness of fit measure (Δ) when excluding each study. Specifically, we can remove signatures associated with each prior study and measure the resulting change in the goodness of fit measure. The larger this change for a study, the more those empirical signatures are inconsistent with the estimated meta-model. This measure may point to studies that are inconsistent with the rest of prior empirical evidence. It may also point to the weaknesses of the meta-model in covering a range of empirical regularities identified in that subset of prior studies.

In our example, we calculate and report these changes (Δ values) in **Table H**, along with this measure normalized by the number of signatures each prior study includes, to have a number that is comparable across prior studies. Based on this measure, we find 3 (out of 16) studies—[52],

[48], and [53]—which together account for 80.1% of the goodness of fit measure. These three studies also include significantly larger error per signature (ranging between 1.3-4.2% per signature, compared to 0.1-0.5% per signature for all other studies). Two of these studies, [48] and [53], are coming from extremely obese subjects (average BMI of 41.6 and 47.1) which may partly explain the larger errors. In fact, in this BMI range, we had limited data from NHANES and thus had lower confidence in the estimated covariance matrix for generation of explanatory variables.

C.6-Minimum theoretical error

To put the comparison of prediction errors on the validation dataset in perspective, it is helpful to have an estimate for the minimum error that is feasible to achieve in predicting BMR using the available explanatory variables (i.e., H, A, F, and L). This theoretical minimum should exist because due to unobserved genetic and environmental factors, individuals would always have some variation in their BMR that is not predictable by the four explanatory variables above. This minimum error would provide us with an estimate for what a perfect model can achieve. We use two approximations to measure this theoretical minimum.

First, we can use the best fitting model structure found using the GMA (i.e., model 4 in **Table I**), and use the validation dataset to estimate the parameters of that model structure. The resulting equation would have the minimum error feasible for the validation sample and provides one approximation for the minimum theoretical error possible. This is the error that is reported in the text (mean absolute percentage error (MAPE) of 6.60%). Note that this method is both estimating and calculating the error using the same sample of 159 subjects, and as such its predictive power may not extend to out of sample predictions. In fact, if we divided our dataset into the training and prediction subsets using a 10-fold cross validation method, the prediction error would increase to 6.73%. This result indicates that GMA, using the results of several large sample studies, can provide equations that are more precise than the one estimated using a smaller sample but the same exact population group.

Alternatively, the minimum error could be approximated using the GMA equation itself. Specifically, the individual variations are accounted for in the error term of the data generating function in our GMA estimate. This error is distributed normally with mean 0 and standard deviation of 136 Kcal/Day in our best fitting model. Therefore, we can generate simulated BMRs (including this noise term) for 159 simulated subjects that have the same values of explanatory variables as those in the validation dataset. We can then compare those simulated BMRs with the predicted values for the same subject, using the GMA-estimated equation. Repeating this procedure for a large number of times (20,000 replications in this case), we can calculate the expected MAPE on this validation dataset, if the data generating process was indeed following our estimated GMA. This expected error is 6.40%, which provides another estimate for the minimum error feasible in predicting BMR.

Either of these estimates for the minimum achievable error—based on the validation dataset—in predicting BMR is very close to the error achieved by GMA, and shows that GMA has closed most of the prediction gap between prior models and the best that is feasible.

Note D- Statistical setup and assumptions of GMA

Recall the general data generating process $y = f(\mathbf{x}, \varepsilon; \boldsymbol{\beta}_0)$ describing the relationship between the response variable y and explanatory variables $\mathbf{x} = [x_i]; \ i = 1,2,...,I$ where $\boldsymbol{\beta}_0 = \lfloor \beta_{0,j} \rfloor; \ j = 1,2,...,J$ are the function's parameters and ε is a random error term with $E(\varepsilon) = 0$.

As mentioned earlier, in GMA, we estimate the data generating model by utilizing the results of L available prior studies, each including a subset of $I_l \leq I; \ l = 1,2,...,L$ explanatory variables and a response variable denoted by $\mathbf{x}_l$ and y_l, respectively. Let $\tilde{\boldsymbol{\gamma}}_l = \mathbf{h}_l(\mathbf{X}_l, \mathbf{y}_l)$ be the empirical signatures from study *l* estimated by n_l observations.

To estimate $\boldsymbol{\beta}_0$ using the GMA, we simulate the function *f* using a set of parameters $\boldsymbol{\beta}$ to generate $\mathbf{y}^s$ values given simulated $\mathbf{X}^s$ values, and transform $\mathbf{X}^s$ and $\mathbf{y}^s$ values using the function $\boldsymbol{\gamma}_l^s(\boldsymbol{\beta}) = \mathbf{h}_l(\mathbf{X}_l^s, \mathbf{y}_l^s(\boldsymbol{\beta}))$. We posit that the resulting vector of statistics ($\boldsymbol{\gamma}_l^s(\boldsymbol{\beta})$) would be close to the estimated parameters in the empirical studies ($\tilde{\boldsymbol{\gamma}}_l$) if $\boldsymbol{\beta}$ is close to $\boldsymbol{\beta}_0$. Consequently, we find a vector of $\boldsymbol{\beta}$ that minimizes the estimated expected error between simulated signatures and empirical signatures, i.e., $\mathbf{e}_l = \frac{1}{S}\sum_{s=1}^{S}(\tilde{\boldsymbol{\gamma}}_l - \boldsymbol{\gamma}_l^s(\boldsymbol{\beta}))$, for each study using the following optimization model.

$$\hat{\boldsymbol{\beta}} = \arg\min_{\boldsymbol{\beta}}\{\mathbf{e}^T \mathbf{W} \mathbf{e}\},$$

where $\mathbf{W}$ is a positive semi-definite matrix and $\mathbf{e} = [\mathbf{e}_0^T \quad \mathbf{e}_1^T \quad \cdots \quad \mathbf{e}_L^T]^T$.

Assumptions:

Let $\boldsymbol{\gamma}_{0,l}$ denote the vector of parameters in study *l*, estimated by the estimator function $\mathbf{h}_l(\mathbf{X}_l, \mathbf{y}_l)$. Suppose the function $\mathbf{h}_l(\mathbf{X}_l, \mathbf{y}_l)$, is in a form of $\tilde{\boldsymbol{\gamma}}_l = \arg\min_{\boldsymbol{\gamma}} q_l(\mathbf{X}_l, \mathbf{y}_l, \boldsymbol{\gamma})$, where $q_l(\mathbf{X}_l, \mathbf{y}_l, \boldsymbol{\gamma})$ can be a negative-likelihood function, a loss function, or any other functions minimized to obtain the estimates. For example, in the case of linear regression, the *q* function for regression coefficients is an ordinary least square loss function. Also, define the vectors of estimated and simulated signatures across all studies, respectively, by $\tilde{\boldsymbol{\gamma}}(.) = [\tilde{\boldsymbol{\gamma}}_0^T(.) \quad \tilde{\boldsymbol{\gamma}}_1^T(.) \quad \ldots \quad \tilde{\boldsymbol{\gamma}}_L^T(.)]^T$ and $\boldsymbol{\gamma}^s(.) = [\boldsymbol{\gamma}_0^{s^T}(.) \quad \boldsymbol{\gamma}_1^{s^T}(.) \quad \ldots \quad \boldsymbol{\gamma}_L^{s^T}(.)]^T$. Following Gourieroux et al. (1993), we make the subsequent assumptions to study the asymptotic properties of proposed GMA.

(A1). The Stochastic loss function used for estimating signatures in study *l*, i.e., $q_l(\mathbf{X}_l, \mathbf{y}_l, \boldsymbol{\gamma})$, asymptotically approaches a continuous non-stochastic function with a unique minimum. For large enough samples, the loss function of each study will be deterministic whose minimizer is the vector of the true parameters of the sub-model estimated in the corresponding study. More rigorously, the vector-valued function

$\mathbf{q}(\mathbf{X},\mathbf{y},\boldsymbol{\gamma}) = [q_0(\mathbf{X}_1,\mathbf{y}_1,\boldsymbol{\gamma}) \quad q_1(\mathbf{X}_2,\mathbf{y}_2,\boldsymbol{\gamma}) \quad \ldots \quad q_l(\mathbf{X}_l,\mathbf{y}_l,\boldsymbol{\gamma})]^T$ asymptotically tends to the non-stochastic function $\mathbf{Q}(\mathbf{F},\mathbf{R},\boldsymbol{\beta}_0,\boldsymbol{\gamma}) = [Q_0(\mathbf{F}_1,\mathbf{R}_1,\boldsymbol{\beta}_0,\boldsymbol{\gamma}) \quad Q_1(\mathbf{F}_2,\mathbf{R}_2,\boldsymbol{\beta}_0,\boldsymbol{\gamma}) \quad \ldots \quad Q_l(\mathbf{F}_l,\mathbf{R}_l,\boldsymbol{\beta}_0,\boldsymbol{\gamma})]^T$ where $Q_l(\mathbf{F}_l,\mathbf{R}_l,\boldsymbol{\beta}_0,\boldsymbol{\gamma})$ is the limit function, and $\mathbf{F}_l$ and $\mathbf{R}_l; l>0$ are marginal distributions of $\mathbf{X}_l$ and $\boldsymbol{\varepsilon}_l$ for study *l*, respectively, and is $\mathbf{R}_0$ the marginal distribution of between-study deviations, assumed to be independent of within-study errors. In other words, $\lim_{n_l\to\infty} \mathbf{q}(\mathbf{X}_l,\mathbf{y}_l,\boldsymbol{\gamma}) = \mathbf{Q}(\mathbf{F},\mathbf{R},\boldsymbol{\beta}_0,\boldsymbol{\gamma})$. We assume that the limit function is continuous and has a unique optimum denoted by $\boldsymbol{\gamma}_0 = \arg\min_{\gamma} \mathbf{Q}(\mathbf{F},\mathbf{R},\boldsymbol{\beta}_0,\boldsymbol{\gamma})$, implying that $\tilde{\boldsymbol{\gamma}}(.)$ is a consistent estimator of $\boldsymbol{\gamma}_0$.

(A2). The information about explanatory variables (i.e., $\mathbf{X}$) given by prior studies suffice to fully identify the distribution of $\mathbf{X}$. This information could be in the form of full distribution information, estimated parameters of the distribution, or observed explanatory variables. In the case that prior studies provide only the estimated parameters of the distribution, it is assumed that those estimates are consistent.

(A3). Define the so-called binding function by $\mathbf{g}(\mathbf{F},\mathbf{R},\cdot) = \arg\min_{\gamma} \mathbf{Q}(\mathbf{F},\mathbf{R},\cdot,\boldsymbol{\gamma})$ and assume $\mathbf{g}(\mathbf{F},\mathbf{R},\cdot)$ is one to one and $\frac{\partial \mathbf{g}(\mathbf{F},\mathbf{R},\boldsymbol{\beta})}{\partial \boldsymbol{\beta}}$ is of full-column rank. Consequently, $\boldsymbol{\gamma}_0 = \mathbf{g}(\mathbf{F},\mathbf{R},\boldsymbol{\beta}_0)$. This assumption states that there exists a minimizer for the limit loss functions of all studies that is defined by marginal distributions of explanatory and response variables. If true parameters of the data generating process ($\boldsymbol{\beta}_0$) were known, this minimizer would be the true parameters of the sub-model in each study.

(A4). Assume the difference between each study's score function and its corresponding simulated score function, i.e., $\tau_l = \sqrt{n_l}\,\frac{\partial q_l(\mathbf{X}_l,\mathbf{y}_l,\boldsymbol{\gamma}_0)}{\partial \boldsymbol{\gamma}} - \sqrt{n_l}\,\frac{1}{S}\sum_{s=1}^{S}\frac{\partial q_l(\mathbf{X}_l^s,\mathbf{y}_l^s,\boldsymbol{\gamma}_0)}{\partial \boldsymbol{\gamma}}$ asymptotically follows a normal distribution with zero mean and a variance given by $\mathbf{V} = \lim_{n_l\to\infty}(\mathrm{var}(\tau_l))$.

Note that these general assumptions discussed in (A1-A4) hold true for most estimators including maximum-likelihood estimators, least square estimators, etc.

Note E- Consistency of GMA estimators

Proposition 1. Under assumptions (A1) - (A3), the GMA estimator $\hat{\boldsymbol{\beta}} = \arg\min_{\boldsymbol{\beta}}\{\mathbf{e}^T\mathbf{W}\mathbf{e}\}$ is a consistent estimator of the data generating model's parameters, $\boldsymbol{\beta}_0$.

Proof: The first-order condition of the optimization criterion, $\min_{\boldsymbol{\beta}}\left[\frac{1}{S}\sum_{s=1}^{S}(\tilde{\boldsymbol{\gamma}}-\boldsymbol{\gamma}^{s}(\boldsymbol{\beta}))\right]^{T}\hat{\mathbf{W}}\left[\frac{1}{S}\sum_{s=1}^{S}(\tilde{\boldsymbol{\gamma}}-\boldsymbol{\gamma}^{s}(\boldsymbol{\beta}))\right]$, is written as $\left[\frac{1}{S}\sum_{s=1}^{S}\frac{\partial\boldsymbol{\gamma}^{s}(\hat{\boldsymbol{\beta}})}{\partial\boldsymbol{\beta}}\right]\hat{\mathbf{W}}\left[\frac{1}{S}\sum_{s=1}^{S}(\tilde{\boldsymbol{\gamma}}-\boldsymbol{\gamma}^{s}(\hat{\boldsymbol{\beta}}))\right]=0$

.

Also, the first-order Taylor expansion of $\left[\frac{1}{S}\sum_{s=1}^{S}(\tilde{\boldsymbol{\gamma}}-\boldsymbol{\gamma}^{s}(\hat{\boldsymbol{\beta}}))\right]$ around $\boldsymbol{\beta}_0$ is given by

$\left[\frac{1}{S}\sum_{s=1}^{S}\frac{\partial\boldsymbol{\gamma}^{s}(\hat{\boldsymbol{\beta}})}{\partial\boldsymbol{\beta}}\right]^{T}\hat{\mathbf{W}}\left[\frac{1}{S}\sum_{s=1}^{S}\left(\tilde{\boldsymbol{\gamma}}-\boldsymbol{\gamma}^{s}(\boldsymbol{\beta}_0)-\frac{\partial\boldsymbol{\gamma}^{s}(\boldsymbol{\beta}_0)}{\partial\boldsymbol{\beta}}(\hat{\boldsymbol{\beta}}-\boldsymbol{\beta}_0)\right)\right]\approx 0$, which leads to

$$(\hat{\boldsymbol{\beta}}-\boldsymbol{\beta}_0)\approx\left(\left[\frac{1}{S}\sum_{s=1}^{S}\frac{\partial\boldsymbol{\gamma}^{s}(\hat{\boldsymbol{\beta}})}{\partial\boldsymbol{\beta}}\right]^{T}\hat{\mathbf{W}}\left[\frac{1}{S}\sum_{s=1}^{S}\frac{\partial\boldsymbol{\gamma}^{s}(\boldsymbol{\beta}_0)}{\partial\boldsymbol{\beta}}\right]\right)^{-1}\left[\frac{1}{S}\sum_{s=1}^{S}\frac{\partial\boldsymbol{\gamma}^{s}(\hat{\boldsymbol{\beta}})}{\partial\boldsymbol{\beta}}\right]^{T}\hat{\mathbf{W}}\left[\tilde{\boldsymbol{\gamma}}-\frac{1}{S}\sum_{s=1}^{S}\boldsymbol{\gamma}^{s}(\boldsymbol{\beta}_0)\right] \qquad \text{(P1)}$$

Recall that $\boldsymbol{\gamma}^{s}(\cdot)=\arg\min_{\boldsymbol{\gamma}} q_l(\mathbf{X}_l^s,\mathbf{y}_l^s,\boldsymbol{\gamma})$ which asymptotically tends to the binding function $\mathbf{g}(\mathbf{F},\mathbf{R},\cdot)$. Consequently, $\boldsymbol{\gamma}^{s}(\boldsymbol{\beta}_0)$ tends to $\mathbf{g}(\mathbf{F},\mathbf{R},\boldsymbol{\beta}_0)=\boldsymbol{\gamma}_0$ (A3). Since $\tilde{\boldsymbol{\gamma}}$ is a consistent estimator of $\boldsymbol{\gamma}_0$ (A1), $\left[\tilde{\boldsymbol{\gamma}}-\frac{1}{S}\sum_{s=1}^{S}\boldsymbol{\gamma}^{s}(\boldsymbol{\beta}_0)\right]\to 0$ for fixed *S*, which implies that $\hat{\boldsymbol{\beta}}$ is a consistent estimator of $\boldsymbol{\beta}_0$. ∎

Note F- Asymptotic distribution of estimators

Proposition 2. Under assumptions (A1)-(A4), the estimator $\hat{\boldsymbol{\beta}}=\arg\min_{\boldsymbol{\beta}}\{\mathbf{e}^{T}\mathbf{W}\mathbf{e}\}$ is asymptotically normal when and *S* is fixed, i.e., $\sqrt{n}\left(\hat{\boldsymbol{\beta}}-\boldsymbol{\beta}_0\right)\xrightarrow{d}N(0,\boldsymbol{\Sigma}(S,\mathbf{W}))$,

where $n=\min\{n_l\}$ and

$$\boldsymbol{\Sigma}(S,\mathbf{W})=\left(1+\frac{1}{S}\right)\left(\frac{\partial\mathbf{g}^{T}(\mathbf{F},\mathbf{R},\boldsymbol{\beta}_0)}{\partial\boldsymbol{\beta}}\mathbf{W}\frac{\partial\mathbf{g}(\mathbf{F},\mathbf{R},\boldsymbol{\beta}_0)}{\partial\boldsymbol{\beta}}\right)^{-1}$$

$$\left(\frac{\partial\mathbf{g}^{T}(\mathbf{F},\mathbf{R},\boldsymbol{\beta}_0)}{\partial\boldsymbol{\beta}}\mathbf{W}\mathbf{J}_0^{-1}(\mathbf{I}_0-\mathbf{K}_0)\mathbf{J}_0^{-1}\mathbf{W}\frac{\partial\mathbf{g}(\mathbf{F},\mathbf{R},\boldsymbol{\beta}_0)}{\partial\boldsymbol{\beta}}\right)\left(\frac{\partial\mathbf{g}^{T}(\mathbf{F},\mathbf{R},\boldsymbol{\beta}_0)}{\partial\boldsymbol{\beta}}\mathbf{W}\frac{\partial\mathbf{g}(\mathbf{F},\mathbf{R},\boldsymbol{\beta}_0)}{\partial\boldsymbol{\beta}}\right)^{-1}$$

where $\mathbf{I}_0=\lim_{n\to\infty}\mathrm{var}\left\{\sqrt{n}\,\frac{\partial\mathbf{q}(\mathbf{X}^s,\mathbf{y}^s,\boldsymbol{\gamma}_0)}{\partial\boldsymbol{\gamma}}\right\}$,

$\mathbf{K}_0=\lim_{n\to\infty}\mathrm{cov}\left\{\sqrt{n}\,\frac{\partial\mathbf{q}(\mathbf{X}^s,\mathbf{y}^s,\boldsymbol{\gamma}_0)}{\partial\boldsymbol{\gamma}},\sqrt{n}\,\frac{\partial\mathbf{q}(\mathbf{X}^r,\mathbf{y}^r,\boldsymbol{\gamma}_0)}{\partial\boldsymbol{\gamma}}\right\}, r\neq s,$ and $\mathbf{J}_0=-\frac{\partial^2\mathbf{Q}(\mathbf{F},\mathbf{R},\boldsymbol{\beta}_0,\boldsymbol{\gamma}_0)}{\partial\boldsymbol{\gamma}\partial\boldsymbol{\gamma}^{T}}$.

Proof: Based on (A1), it is true that $\frac{\partial \mathbf{q}(\mathbf{X},\mathbf{y},\tilde{\boldsymbol{\gamma}})}{\partial \boldsymbol{\gamma}} = 0$, where $\frac{\partial \mathbf{q}(\mathbf{X},\mathbf{y},\tilde{\boldsymbol{\gamma}})}{\partial \boldsymbol{\gamma}}$ denotes $\frac{\partial \mathbf{q}(\mathbf{X},\mathbf{y},\boldsymbol{\gamma})}{\partial \boldsymbol{\gamma}}$ evaluated at $\tilde{\boldsymbol{\gamma}}$. Using the first-order Taylor expansion of $\frac{\partial \mathbf{q}(\mathbf{X},\mathbf{y},\tilde{\boldsymbol{\gamma}})}{\partial \boldsymbol{\gamma}}$ around $\boldsymbol{\gamma}_0$, we get $\frac{\partial \mathbf{q}(\mathbf{X},\mathbf{y},\boldsymbol{\gamma}_0)}{\partial \boldsymbol{\gamma}} + \frac{\partial^2 \mathbf{q}(\mathbf{X},\mathbf{y},\boldsymbol{\gamma}_0)}{\partial \boldsymbol{\gamma}\partial \boldsymbol{\gamma}^T}(\tilde{\boldsymbol{\gamma}} - \boldsymbol{\gamma}_0) \approx 0$. Thus, $(\tilde{\boldsymbol{\gamma}} - \boldsymbol{\gamma}_0) \approx \mathbf{J}_0^{-1} \frac{\partial \mathbf{q}(\mathbf{X},\mathbf{y},\boldsymbol{\gamma}_0)}{\partial \boldsymbol{\gamma}}$.

Consider the dataset used in each study (i.e., $\mathbf{X}_l$ and $\mathbf{y}_l$) as one stream of simulated data, denoted by $\mathbf{X}^s$ and $\mathbf{y}^s$. Consequently, by replacing $\tilde{\boldsymbol{\gamma}}$ with $\boldsymbol{\gamma}^s(\boldsymbol{\beta})$, we have

$$(\boldsymbol{\gamma}^s(\boldsymbol{\beta}) - \boldsymbol{\gamma}_0) \approx \mathbf{J}_0^{-1} \frac{\partial \mathbf{q}(\mathbf{X}^s,\mathbf{y}^s,\boldsymbol{\gamma}_0)}{\partial \boldsymbol{\gamma}}.$$

Thus, $\sqrt{n}(\tilde{\boldsymbol{\gamma}} - \frac{1}{S}\sum_{s=1}^{S}\boldsymbol{\gamma}^s(\boldsymbol{\beta})) \approx \mathbf{J}_0^{-1}\left\{\sqrt{n}\frac{\partial \mathbf{q}(\mathbf{X},\mathbf{y},\boldsymbol{\gamma}_0)}{\partial \boldsymbol{\gamma}} - \sqrt{n}\frac{1}{S}\sum_{s=1}^{S}\frac{\partial \mathbf{q}(\mathbf{X}^s,\mathbf{y}^s,\boldsymbol{\gamma}_0)}{\partial \boldsymbol{\gamma}}\right\}$. Therefore, under assumptions (A4), this difference is asymptotically normally distributed with zero mean and the following covariance matrix.

$$\operatorname{var}\left(\sqrt{n}(\tilde{\boldsymbol{\gamma}} - \frac{1}{S}\sum_{s=1}^{S}\boldsymbol{\gamma}^s(\boldsymbol{\beta}))\right) = \mathbf{J}_0^{-1}\left\{\left(1+\frac{1}{S}\right)\mathbf{I}_0 - 2\mathbf{K}_0 + \frac{S(S-1)}{S^2}\mathbf{K}_0\right\}\mathbf{J}_0^{-1} = \left(1+\frac{1}{S}\right)\mathbf{J}_0^{-1}(\mathbf{I}_0 - \mathbf{K}_0)\mathbf{J}_0^{-1}. \quad \text{(P2)}$$

Based on (A2) and (A3), (P1) in Proposition S2, and the fact that $\partial\boldsymbol{\gamma}^s(\hat{\boldsymbol{\beta}})$ asymptotically tends to $\boldsymbol{\gamma}_0 = \mathbf{g}(\mathbf{F},\mathbf{R},\boldsymbol{\beta}_0)$,

$$\sqrt{n}(\hat{\boldsymbol{\beta}} - \boldsymbol{\beta}_0) \approx \left(\frac{\partial \mathbf{g}^T(\mathbf{F},\mathbf{R},\boldsymbol{\beta}_0)}{\partial \boldsymbol{\beta}}\mathbf{W}\frac{\partial \mathbf{g}(\mathbf{F},\mathbf{R},\boldsymbol{\beta}_0)}{\partial \boldsymbol{\beta}}\right)^{-1}\frac{\partial \mathbf{g}^T(\mathbf{F},\mathbf{R},\boldsymbol{\beta}_0)}{\partial \boldsymbol{\beta}}\mathbf{W}\sqrt{n}\left[\tilde{\boldsymbol{\gamma}} - \frac{1}{S}\sum_{s=1}^{S}\boldsymbol{\gamma}^s(\boldsymbol{\beta}_0)\right].$$

Consequently, $\sqrt{n}(\hat{\boldsymbol{\beta}} - \boldsymbol{\beta}_0)$ is asymptotically normally distributed with zero mean and the variance of

$$\left(\frac{\partial \mathbf{g}^T(\mathbf{F},\mathbf{R},\boldsymbol{\beta}_0)}{\partial \boldsymbol{\beta}}\mathbf{W}\frac{\partial \mathbf{g}(\mathbf{F},\mathbf{R},\boldsymbol{\beta}_0)}{\partial \boldsymbol{\beta}}\right)^{-1}\left(\frac{\partial \mathbf{g}^T(\mathbf{F},\mathbf{R},\boldsymbol{\beta}_0)}{\partial \boldsymbol{\beta}}\mathbf{W}\operatorname{var}\left(\sqrt{n}\left[\tilde{\boldsymbol{\gamma}} - \frac{1}{S}\sum_{s=1}^{S}\boldsymbol{\gamma}^s(\boldsymbol{\beta}_0)\right]\right)\mathbf{W}\frac{\partial \mathbf{g}(\mathbf{F},\mathbf{R},\boldsymbol{\beta}_0)}{\partial \boldsymbol{\beta}}\right).$$
$$\left(\frac{\partial \mathbf{g}^T(\mathbf{F},\mathbf{R},\boldsymbol{\beta}_0)}{\partial \boldsymbol{\beta}}\mathbf{W}\frac{\partial \mathbf{g}(\mathbf{F},\mathbf{R},\boldsymbol{\beta}_0)}{\partial \boldsymbol{\beta}}\right)$$

Using (P2), the asymptotic variance of $\sqrt{n}(\hat{\boldsymbol{\beta}} - \boldsymbol{\beta}_0)$ is written as

$$\boldsymbol{\Sigma}(S,\mathbf{W}) = \left(1+\frac{1}{S}\right)\left(\frac{\partial \mathbf{g}^T(\mathbf{F},\mathbf{R},\boldsymbol{\beta}_0)}{\partial \boldsymbol{\beta}}\mathbf{W}\frac{\partial \mathbf{g}(\mathbf{F},\mathbf{R},\boldsymbol{\beta}_0)}{\partial \boldsymbol{\beta}}\right)^{-1}$$

$$\left(\frac{\partial \mathbf{g}^T(\mathbf{F},\mathbf{R},\boldsymbol{\beta}_0)}{\partial \boldsymbol{\beta}}\mathbf{W}\mathbf{J}_0^{-1}(\mathbf{I}_0 - \mathbf{K}_0)\mathbf{J}_0^{-1}\mathbf{W}\frac{\partial \mathbf{g}(\mathbf{F},\mathbf{R},\boldsymbol{\beta}_0)}{\partial \boldsymbol{\beta}}\right)\left(\frac{\partial \mathbf{g}^T(\mathbf{F},\mathbf{R},\boldsymbol{\beta}_0)}{\partial \boldsymbol{\beta}}\mathbf{W}\frac{\partial \mathbf{g}(\mathbf{F},\mathbf{R},\boldsymbol{\beta}_0)}{\partial \boldsymbol{\beta}}\right)^{-1}. \blacksquare$$

Note G- Optimal choice of signatures' covariance matrix and its estimate

Proposition 3. Under assumptions (A1)-(A4), the optimal choice of the matrix $\mathbf{W}$ is $\mathbf{W}^* = \mathbf{J}_0^{-1}(\mathbf{I}_0 - \mathbf{K}_0)\mathbf{J}_0^{-1}$, which minimizes $\mathbf{\Sigma}(S, \mathbf{W})$. Consequently, the optimal $\mathbf{\Sigma}(S, \mathbf{W})$ is $\mathbf{\Sigma}^*(S, \mathbf{W}^*) = \left(1 + \frac{1}{S}\right)\left(\frac{\partial \mathbf{g}^T(\mathbf{F}, \mathbf{R}, \boldsymbol{\beta}_0)}{\partial \boldsymbol{\beta}} \mathbf{J}_0 (\mathbf{I}_0 - \mathbf{K}_0)^{-1} \mathbf{J}_0 \frac{\partial \mathbf{g}(\mathbf{F}, \mathbf{R}, \boldsymbol{\beta}_0)}{\partial \boldsymbol{\beta}}\right)^{-1}$.

A consistent estimator of $\mathbf{\Sigma}(S, \mathbf{W})$ is given by $\hat{\mathbf{\Sigma}}^*(S, \mathbf{W}^*) = \left(1 + \frac{1}{S}\right)\left(\mathbf{\Gamma}^T \mathbf{\Delta}^{-1} \mathbf{\Gamma}\right)^{-1}$ where $\mathbf{\Delta} = \frac{1}{S}\sqrt{n}\sum_{s=1}^{S}(\mathbf{M}_s - \overline{\mathbf{M}})(\mathbf{M}_s - \overline{\mathbf{M}})^T$ with $\mathbf{M}_s = \frac{\partial \mathbf{q}(\mathbf{X}^s, \mathbf{y}^s, \tilde{\boldsymbol{\gamma}})}{\partial \boldsymbol{\gamma}}$; $\overline{\mathbf{M}} = \frac{1}{S}\sum_{s=1}^{S}\mathbf{M}_s$; and $\mathbf{X}^s$ and $\mathbf{y}^s$ are generated using any consistent estimator of $\boldsymbol{\beta}_0$, e.g., $\hat{\boldsymbol{\beta}}$ computed using an identity $\mathbf{W}$ matrix. $\mathbf{\Gamma}$ is a numerical derivation of $\mathbf{M}_s$ with respect to $\boldsymbol{\beta}$ evaluated at $\hat{\boldsymbol{\beta}}$.

Proof: As shown in Gourieroux et al. [18], $\mathbf{\Delta}$ is a consistent estimator of $\mathbf{W}^* = (\mathbf{I}_0 - \mathbf{K}_0)$. Also, as $\lim_{\mathbf{n}\to\infty} -\frac{\partial^2 \mathbf{q}(\mathbf{X}^s, \mathbf{y}^s, \tilde{\boldsymbol{\gamma}})}{\partial \boldsymbol{\gamma} \partial \boldsymbol{\gamma}^T} = -\frac{\partial^2 \mathbf{Q}(\mathbf{F}, \mathbf{R}, \boldsymbol{\beta}_0, \boldsymbol{\gamma})}{\partial \boldsymbol{\gamma} \partial \boldsymbol{\gamma}^T} = \mathbf{J}_0$, a consistent estimator of $\mathbf{J}_0$ is given by $-\frac{\partial^2 \mathbf{q}(\mathbf{X}^s, \mathbf{y}^s, \tilde{\boldsymbol{\gamma}})}{\partial \boldsymbol{\gamma} \partial \boldsymbol{\gamma}^T}$.

Recall that $\boldsymbol{\gamma}_0 = \arg\min_{\gamma} \mathbf{Q}(\mathbf{F}, \mathbf{R}, \boldsymbol{\beta}_0, \boldsymbol{\gamma}) = \mathbf{g}(\mathbf{F}, \mathbf{R}, \boldsymbol{\beta}_0)$. Therefore, the first-order condition is written as $\frac{\partial \mathbf{Q}(\mathbf{F}, \mathbf{R}, \boldsymbol{\beta}, \mathbf{g}(\mathbf{F}, \mathbf{R}, \boldsymbol{\beta}))}{\partial \boldsymbol{\gamma}} = 0$. Consequently, the second derivative with respect to $\boldsymbol{\beta}$ gives $\frac{\partial^2 \mathbf{Q}(\mathbf{F}, \mathbf{R}, \boldsymbol{\beta}_0, \boldsymbol{\gamma}_0)}{\partial \boldsymbol{\gamma} \partial \boldsymbol{\beta}^T} + \frac{\partial^2 \mathbf{Q}(\mathbf{F}, \mathbf{R}, \boldsymbol{\beta}_0, \boldsymbol{\gamma}_0)}{\partial \boldsymbol{\gamma} \partial \boldsymbol{\gamma}^T} \frac{\partial \mathbf{g}(\mathbf{F}, \mathbf{R}, \boldsymbol{\beta}_0)}{\partial \boldsymbol{\beta}} = 0$, which implies $\frac{\partial \mathbf{g}(\mathbf{F}, \mathbf{R}, \boldsymbol{\beta}_0)}{\partial \boldsymbol{\beta}} = \mathbf{J}_0^{-1} \frac{\partial^2 \mathbf{Q}(\mathbf{F}, \mathbf{R}, \boldsymbol{\beta}_0, \boldsymbol{\gamma}_0)}{\partial \boldsymbol{\gamma} \partial \boldsymbol{\beta}^T}$.

Replacing $(\mathbf{I}_0 - \mathbf{K}_0)$, $\mathbf{J}_0$ and $\frac{\partial^2 \mathbf{Q}(\mathbf{F}, \mathbf{R}, \boldsymbol{\beta}_0, \boldsymbol{\gamma}_0)}{\partial \boldsymbol{\gamma} \partial \boldsymbol{\beta}^T}$ with their consistent estimators leads to a consistent estimator for $\mathbf{\Sigma}(S, \mathbf{W})$ given by

$$\hat{\mathbf{\Sigma}}^*(S, \mathbf{W}^*) = \left(1 + \frac{1}{S}\right)\left(\mathbf{\Gamma}^T \mathbf{\Delta}^{-1} \mathbf{\Gamma}\right)^{-1}$$

In practice, we start with a promising W (e.g., the diagonal matrix with reciprocal of squared signatures), estimate the $\hat{\boldsymbol{\beta}}$, then use that to simulate a large sample (in 1000s) of signatures and calculate the simulated covariance matrix, which will be used in the second iteration to estimate $\hat{\boldsymbol{\beta}}$. The process can be continued until convergence, which typically happens in a handful of iterations.

Note H- Asymptotic variance of meta-model estimate using delta method

As shown in S3, under assumptions (A1)-(A4),

$$\sqrt{n}\left(\hat{\boldsymbol{\beta}}-\boldsymbol{\beta}\right)\xrightarrow{d}N(0,\boldsymbol{\Sigma}(S,\mathbf{W}))\ .$$

Estimation of $\boldsymbol{\Sigma}(S,\mathbf{W})$, however, could be cumbersome, especially when $\mathbf{q}(\cdot)$ functions have nonlinear and complex forms. In such a case, one can use the Delta method (Bishop et al., 1973, Section 14.6) to approximate the covariance function.

Suppose $\hat{\boldsymbol{\beta}}=\arg\min_{\boldsymbol{\beta}}\left[\frac{1}{S}\sum_{s=1}^{S}(\tilde{\boldsymbol{\gamma}}-\boldsymbol{\gamma}^s(\boldsymbol{\beta}))\right]^T\mathbf{W}\left[\frac{1}{S}\sum_{s=1}^{S}(\tilde{\boldsymbol{\gamma}}-\boldsymbol{\gamma}^s(\boldsymbol{\beta}))\right]$ and $\boldsymbol{\Sigma}_0$ denote the asymptotic covariance of $\left(\frac{1}{S}\sum_{s=1}^{S}(\tilde{\boldsymbol{\gamma}}-\boldsymbol{\gamma}^s(\boldsymbol{\beta}))\right)$. Using the Delta method the asymptotic covariance of $\hat{\boldsymbol{\beta}}$ is written as

$$\tilde{\boldsymbol{\Sigma}}=\nabla\left(\left[\frac{1}{S}\sum_{s=1}^{S}(\tilde{\boldsymbol{\gamma}}-\boldsymbol{\gamma}^s(\boldsymbol{\beta}))\right]^T\mathbf{W}\left[\frac{1}{S}\sum_{s=1}^{S}(\tilde{\boldsymbol{\gamma}}-\boldsymbol{\gamma}^s(\boldsymbol{\beta}))\right]\right)^T\boldsymbol{\Sigma}_0\nabla\left(\left[\frac{1}{S}\sum_{s=1}^{S}(\tilde{\boldsymbol{\gamma}}-\boldsymbol{\gamma}^s(\boldsymbol{\beta}))\right]^T\mathbf{W}\left[\frac{1}{S}\sum_{s=1}^{S}(\tilde{\boldsymbol{\gamma}}-\boldsymbol{\gamma}^s(\boldsymbol{\beta}))\right]\right)$$

After algebraic simplification, we have

$$\tilde{\boldsymbol{\Sigma}}=(1+\frac{1}{S})\left(\left[\frac{1}{S}\sum_{s=1}^{S}\frac{\partial\boldsymbol{\gamma}^s(\hat{\boldsymbol{\beta}})}{\partial\boldsymbol{\beta}}\right]^T\mathbf{W}\left[\frac{1}{S}\sum_{s=1}^{S}\frac{\partial\boldsymbol{\gamma}^s(\hat{\boldsymbol{\beta}})}{\partial\boldsymbol{\beta}}\right]\right)^{-1}\left[\frac{1}{S}\sum_{s=1}^{S}\frac{\partial\boldsymbol{\gamma}^s(\hat{\boldsymbol{\beta}})}{\partial\boldsymbol{\beta}}\right]^T\mathbf{W}\boldsymbol{\Sigma}_0\mathbf{W}\times$$

$$\left[\frac{1}{S}\sum_{s=1}^{S}\frac{\partial\boldsymbol{\gamma}^s(\hat{\boldsymbol{\beta}})}{\partial\boldsymbol{\beta}}\right]\left(\left[\frac{1}{S}\sum_{s=1}^{S}\frac{\partial\boldsymbol{\gamma}^s(\hat{\boldsymbol{\beta}})}{\partial\boldsymbol{\beta}}\right]^T\mathbf{W}\left[\frac{1}{S}\sum_{s=1}^{S}\frac{\partial\boldsymbol{\gamma}^s(\hat{\boldsymbol{\beta}})}{\partial\boldsymbol{\beta}}\right]\right)^{-1}$$

If the optimal weight matrix ($\mathbf{W}^*$), discussed in S4 is used, the approximate covariance matrix can be rewritten as

$$\tilde{\boldsymbol{\Sigma}}=(1+\frac{1}{S})\left(\left[\frac{1}{S}\sum_{s=1}^{S}\frac{\partial\boldsymbol{\gamma}^s(\hat{\boldsymbol{\beta}})}{\partial\boldsymbol{\beta}}\right]^T\mathbf{W}^*\left[\frac{1}{S}\sum_{s=1}^{S}\frac{\partial\boldsymbol{\gamma}^s(\hat{\boldsymbol{\beta}})}{\partial\boldsymbol{\beta}}\right]\right)^{-1}\ .$$

Note I- Confidence intervals using bootstrapping methods

The use of boot-strapping for the calculation of confidence intervals is straight forward. We start by estimating the meta-model using the GMA. Then we generate **M** simulated data sets using the estimated $\hat{\boldsymbol{\beta}}$, conduct the imitations of prior studies on these **M** studies, and then estimate the parameters of each using the GMA. The empirical distributions of the resulting **M** estimates for $\hat{\boldsymbol{\beta}}$ can be used to construct the bootstrap confidence intervals. **Note C.3** provides a comparison of using bootstrapping and analytic confidence intervals in our empirical application.

Note J- Goodness of fit test

Given that the number of signatures is typically larger than the number of parameters for the true function that we estimate, the error function minimized in GMA usually does not reach zero. In fact, the size of this error function provides insights into the goodness of model specification, i.e., how well the estimated model replicates the empirical signatures.

Proposition- Under assumptions (A1)-(A4), if the data generating model is correctly specified, the following statistic is asymptotically distributed as a chi-square with the degrees of freedom equal to $\dim(\boldsymbol{\gamma}) - \dim(\boldsymbol{\beta})$.

$$\chi_0 = \frac{S}{1+S} \min_{\boldsymbol{\beta}} \left[\sqrt{n} \frac{1}{S} \sum_{s=1}^{S} (\tilde{\boldsymbol{\gamma}} - \boldsymbol{\gamma}^s(\boldsymbol{\beta})) \right]^T \hat{\mathbf{W}}^* \left[\frac{1}{S} \sum_{s=1}^{S} (\tilde{\boldsymbol{\gamma}} - \boldsymbol{\gamma}^s(\boldsymbol{\beta})) \right],$$

Where $\hat{\mathbf{W}}^*$ is a consistent estimator of $\mathbf{W}^*$.

Proof: Suppose $\hat{\boldsymbol{\beta}}$ is the GMA estimator. Consequently, the test statistic χ_0 is given by

$$\frac{S}{1+S} \sqrt{n} \left[\frac{1}{S} \sum_{s=1}^{S} (\tilde{\boldsymbol{\gamma}} - \boldsymbol{\gamma}^s(\hat{\boldsymbol{\beta}})) \right]^T \hat{\mathbf{W}}^* \left[\frac{1}{S} \sum_{s=1}^{S} (\tilde{\boldsymbol{\gamma}} - \boldsymbol{\gamma}^s(\hat{\boldsymbol{\beta}})) \right]$$

The first-order Taylor expansion around $\boldsymbol{\beta}_0$ is written as

$$\chi_0 \approx \frac{S}{1+S} \sqrt{n} \left[\frac{1}{S} \sum_{s=1}^{S} \left(\tilde{\boldsymbol{\gamma}} - \boldsymbol{\gamma}^s(\boldsymbol{\beta}_0) - \frac{\partial \gamma^s(\boldsymbol{\beta}_0)}{\partial \boldsymbol{\beta}} (\hat{\boldsymbol{\beta}} - \boldsymbol{\beta}_0) \right) \right]^T \hat{\mathbf{W}}^* \left[\frac{1}{S} \sum_{s=1}^{S} \left(\tilde{\boldsymbol{\gamma}} - \boldsymbol{\gamma}^s(\boldsymbol{\beta}_0) - \frac{\partial \gamma^s(\boldsymbol{\beta}_0)}{\partial \boldsymbol{\beta}} (\hat{\boldsymbol{\beta}} - \boldsymbol{\beta}_0) \right) \right]$$

From P1, it is given that

$$\left[\frac{1}{S} \sum_{s=1}^{S} \frac{\partial \gamma^s(\boldsymbol{\beta}_0)}{\partial \boldsymbol{\beta}} \right] (\hat{\boldsymbol{\beta}} - \boldsymbol{\beta}_0) \approx$$

$$\left[\frac{1}{S} \sum_{s=1}^{S} \frac{\partial \gamma^s(\boldsymbol{\beta}_0)}{\partial \boldsymbol{\beta}} \right] \left(\left[\frac{1}{S} \sum_{s=1}^{S} \frac{\partial \gamma^s(\boldsymbol{\beta}_0)}{\partial \boldsymbol{\beta}} \right]^T \mathbf{W}^* \left[\frac{1}{S} \sum_{s=1}^{S} \frac{\partial \gamma^s(\boldsymbol{\beta}_0)}{\partial \boldsymbol{\beta}} \right] \right)^{-1} \left[\frac{1}{S} \sum_{s=1}^{S} \frac{\partial \gamma^s(\boldsymbol{\beta}_0)}{\partial \boldsymbol{\beta}} \right]^T \mathbf{W}^* \left[\hat{\boldsymbol{\gamma}} - \frac{1}{S} \sum_{s=1}^{S} \boldsymbol{\gamma}^s(\boldsymbol{\beta}_0) \right]$$

and thus,

$$\sqrt{n}\left[\tilde{\boldsymbol{\gamma}}-\frac{1}{S}\sum_{s=1}^{S}\boldsymbol{\gamma}^{s}(\boldsymbol{\beta}_0)\right]-\sqrt{n}\left[\frac{1}{S}\sum_{s=1}^{S}\frac{\partial\boldsymbol{\gamma}^{s}(\boldsymbol{\beta}_0)}{\partial\boldsymbol{\beta}}\right](\hat{\boldsymbol{\beta}}-\boldsymbol{\beta}_0)\approx(\mathbf{I}-\mathbf{R})\sqrt{n}\left[\tilde{\boldsymbol{\gamma}}-\frac{1}{S}\sum_{s=1}^{S}\boldsymbol{\gamma}^{s}(\boldsymbol{\beta}_0)\right],$$

where **R** is an orthogonal idempotent matrix with full-column rank

$$\mathbf{R}=\left[\frac{1}{S}\sum_{s=1}^{S}\frac{\partial\boldsymbol{\gamma}^{s}(\hat{\boldsymbol{\beta}})}{\partial\boldsymbol{\beta}}\right]\left(\left[\frac{1}{S}\sum_{s=1}^{S}\frac{\partial\boldsymbol{\gamma}^{s}(\hat{\boldsymbol{\beta}})}{\partial\boldsymbol{\beta}}\right]^{T}\mathbf{W}^{*}\left[\frac{1}{S}\sum_{s=1}^{S}\frac{\partial\boldsymbol{\gamma}^{s}(\hat{\boldsymbol{\beta}})}{\partial\boldsymbol{\beta}}\right]\right)^{-1}\left[\frac{1}{S}\sum_{s=1}^{S}\frac{\partial\boldsymbol{\gamma}^{s}(\hat{\boldsymbol{\beta}})}{\partial\boldsymbol{\beta}}\right]\mathbf{W}^{*}.$$

Therefore, the test statistic is written as

$$\chi_0\approx\frac{S}{1+S}\sqrt{n}\left[\tilde{\boldsymbol{\gamma}}-\frac{1}{S}\sum_{s=1}^{S}\boldsymbol{\gamma}^{s}(\boldsymbol{\beta}_0)\right]^{T}(\mathbf{I}-\mathbf{R})^{T}\mathbf{W}^{*}(\mathbf{I}-\mathbf{R})\left[\tilde{\boldsymbol{\gamma}}-\frac{1}{S}\sum_{s=1}^{S}\boldsymbol{\gamma}^{s}(\boldsymbol{\beta}_0)\right]$$

From Proposition 2, we know that

$$\sqrt{n}\left[\tilde{\boldsymbol{\gamma}}-\frac{1}{S}\sum_{s=1}^{S}\boldsymbol{\gamma}^{s}(\boldsymbol{\beta}_0)\right]\xrightarrow{d}N\left(0,\left(\frac{S}{1+S}\mathbf{W}^{*}\right)^{-1}\right)$$

Therefore, $\chi_0\xrightarrow{d}\chi^2_{\dim(\boldsymbol{\gamma})-\dim(\boldsymbol{\beta})}$. ■

Note K- Model selection criterion

In reality, the structure of true model *f* is often unknown, and one can only presume the structure based on experience and domain knowledge. Thus, it would be common to propose and estimate a set of candidates for the true model, choosing the one that fits the data better. Intuitively, the goodness-of-fit test statistic (χ_0), introduced earlier, can be used as a criterion for model selection. However, if χ_0 is used as the sole criterion, the metric favors more complex models with more parameters: more complex models are prone to over-fit the signatures and thus have smaller χ_0 values. To avoid overfitting, similar to Akaike information criterion (AIC), we discourage model complexity by introducing a penalty that penalizes the large number of model parameters. Hence, the model selection criterion (MSC) is defined by

$$MSC=\chi_0+\lambda\times\dim(\beta),$$

where $\dim(\beta)$ is the number of model parameters, and $\lambda>0$ is a weight factor promoting model simplicity. Larger values of λ lead to simpler models and vice versa. In AIC, the λ value is chosen to be two. The MSC criterion is calculated for a set of model candidates that pass the "specification test" and the one with the least MSC value is chosen as the best candidate for the true model.

Supplementary Tables

Table A. Comparison of prior studies and meta-model in 1,000 estimations of the first scenario

**Prior models from Scenario 1**		**Parameters**				
		γ_1	γ_2	γ_3	γ_4	γ_5
**Study 1**	Inclusion percentage[1]	94.5%	48.5%	0.0%		0.0%
	Mean (SD)[2]	1.004 (0.190)	1.839 (0.412)	2.551 (0.189)		3.425 (0.494)
	Means of 95% CI bounds	0.634 – 1.374	1.003 – 2.674	2.178 – 2.924		2.414 – 4.230
**Study 2**	Inclusion percentage	95.7%	87.8%		0.1%	36.3%
	Mean (SD)	0.995 (0.123)	0.783 (0.280)		1.321 (0.056)	1.508 (0.210)
	Means of 95% CI bounds	0.749 – 1.240	0.218 – 1.347		1.209 – 1.434	1.063 – 1.862
**Study 3**	Inclusion percentage	96.1%		91.5%	84.7%	87.7%
	Mean (SD)	1.006 (0.113)		0.915 (0.146)	1.063 (0.068)	1.195 (0.172)
	Means of 95% CI bounds	0.787 – 1.224		0.607 – 1.223	0.925 – 1.201	0.843 – 1.475
**Meta-model estimated by GMA**[3]	Inclusion percentage	93.7%	93.2%	89.3%	90.7%	83.5%
	Mean (SD)	1.001 (0.079)	0.967 (0.249)	0.961 (0.147)	1.013 (0.060)	1.016 (0.091)
	Means of 95% CI bounds	0.856 – 1.147	0.516 – 1.418	0.717 – 1.205	0.908 – 1.117	0.881 – 1.151

[1]Percentage of estimated 95% confidence intervals including the true value over 1,000 estimations; [2]Mean and Standard Deviation of 1000 estimations; [3]Covariance for explanatory variables are extracted from prior studies—see **Table C** for true covariance matrix.

Table B. Comparison of meta-models in 1,000 estimations of the first scenario with covariance extracted from prior studies (top row) and unknown correlations estimated using the GMA (bottom row).

**Prior models from Scenario 1**		**Parameters**							
		β_0 true value=1	β_1 true value=1	β_2 true value=1	β_3 true value=1	β_4 true value=1	β_5 true value=0.05	β_6 true value=0.2	β_7 true value=0.7
**Meta-model estimated by GMA***	Inclusion percentage[1]	93.7%	93.2%	89.3%	90.7%	83.5%			
	Mean (SD)[2]	1.001 (0.079)	0.967 (0.249)	0.961 (0.147)	1.013 (0.060)	1.016 (0.091)			
	Means of 95% CI bounds	0.856 – 1.147	0.516 – 1.418	0.717 – 1.205	0.908 – 1.117	0.881 – 1.151			
**Meta-model estimated by GMA†**	Inclusion percentage	93.6%	99.6%	95.5%	99.8%	98.7%	100%	100%	100%
	Mean (SD)	1.003 (0.078)	0.996 (0.259)	0.989 (0.133)	1.004 (0.055)	0.998 (0.089)	0.068 (0.117)	0.209 (0.115)	0.699 (0.061)
	Means of 95% CI bounds	0.855 – 1.150	0.103 – 1.889	0.612 – 1.366	0.888 – 1.119	0.749 – 1.247	-0.708 – 0.845	-0.435 – 0.853	0.530 – 0.864

[1]Percentage of estimated 95% confidence intervals including the true value over 1,000 estimations; [2]Mean and Standard Deviation of 1000 estimations;

*Covariance for explanatory variables are extracted from prior studies; †Covariance for explanatory variables are estimated— β_{5-7} are the correlations of the explanatory variables.

Table C. Mean and covariance for explanatory variables in the first four scenarios

**Variable**	**Mean**	**Covariance matrix***		
		X_1	X_2	X_3
X_1	0	0.20	0.02	0.20
X_2	0	0.02	1.00	1.57
X_3	0	0.20	1.57	5.00

*Correlation coefficients are: $\rho_{X_1,X_2} = 0.05$, $\rho_{X_1,X_3} = 0.2$, $\rho_{X_2,X_3} = 0.7$

Table D. Results for estimating a continuous underlying model using ANOVA results that assess the mean effects of discretized explanatory variables

ANOVA Experiment		Parameters				
		β_0	β_1	β_2	β_3	β_4
**Sample Study**	GMA Estimate<br>95% Confidence Interval	1.05<br>(0.82,1.29)	0.84<br>(0.33,1.36)	1.09<br>(0.89,1.29)	0.78<br>(-0.18,1.74)	1.05<br>(0.85,1.25)
**80% Confidence Interval**	Inclusion percentage out of 1000 iterations	79.6%	79.3%	80.4%	80.4%	82.9%
**95% Confidence Interval**	Inclusion percentage out of 1000 iterations	95.5%	93.3%	95.3%	92.9%	96.7%

Table E. Non-linear example: measuring leakage of transmission fluid (Y) in power systems based on temperature and time

Underlying true model (non-linear) [*] $F = (\beta_{0_1} + \beta_{0_2}T)(1 - \exp\{-\beta_{0_3}t\}) + \varepsilon_0$	Intercept	Temperature (T)	Time (t)	σ_e	$\bar{R}^2$
	β_1	β_2	β_3	β_4	
	1.014	-0.122	0.675	2.22E-5	
Linear model 1 $F_2 = a_2 + b_2t + cT + \varepsilon_1$	0.981 (0.034)	-0.122 (0.005)	0.012 (0.001)	8.97E-05	0.849
Linear model 2 $F_1 = a_1 + b_1t + \varepsilon_2$	0.086 (0.005) [†]		0.011 (0.002)	8.95E-05	0.491
Meta-model estimated by GMA $F = (\beta_1 + \beta_2T)(1 - \exp\{-\beta_3t\}) + \varepsilon_3$	1.044 (0.027)	-0.126 (0.004)	0.712 (0.061)	1.32E-05 (6.51E-05)	**0.997** [‡]

[*] Underlying true model is extracted from Paynabar et al. (2012), equation (2) for part #11. [†] Standard Deviation. [‡] Model Selection Criterion—smaller value of *MSC* indicates a better fit—see **Note K** for more discussion about the *MSC*. [‡] Goodness of fit test does not reject the meta-model: $(\chi_0 = 3.4) < (\chi^2_{d=3} = 7.8)$.

Table F. Comparison of GMA and Analytical random-effects meta-analysis across five scenarios with different between study standard deviations (σ). Results are averaged over 200 replications in each scenario.

**GMA vs. Analytical**	$\boldsymbol{\sigma^2 = 1}$	$\boldsymbol{\sigma^2 = 2}$	$\boldsymbol{\sigma^2 = 4}$	$\boldsymbol{\sigma^2 = 8}$	$\boldsymbol{\sigma^2 = 16}$
μ GMA MAE*	0.91	0.96	1.11	1.34	1.69
μ Analytical MAE	1.02	1.00	1.11	1.34	1.69
σ^2 GMA MAPE†	**231%**	**170%**	**106%**	90%	73%
σ^2 Analytical MAPE	309%	192%	124%	91%	76%

*Mean absolute error; †Mean absolute percentage error

Table G. Results of measurement error analysis based on the case of scenario one, assuming measurement noise levels as a fraction (δ between 0 and 1) of the underlying variables' actual standard deviations, and estimating the extended meta-model with three additional unknown parameters

**Prior models from Scenario 1**			**Parameters**					Standard deviations of measurement errors for the three explanatory variables ($X_1 – X_3$)[†]		
			β_0 true value=1	β_1 true value=1	β_2 true value=1	β_3 true value=1	β_4 true value=1	β_5 true value= $\delta\sqrt{0.2}$	β_6 true value= $\delta\sqrt{1}$	β_7 true value= $\delta\sqrt{5}$
	$\delta = 0$	Inclusion percentage[1]	96%	92%	90%	89%	100%	82%	82%	85%
		Mean[2]	1.01	2.16	1.46	0.85	0.34	0.17	0.25	0.33
		Median[3]	1.01	1.25	1.17	0.87	0.17	0.14	0.20	0.26
		95% CI bounds[4]	0.88 - 1.16	0.43 - 7.72	0.35 - 3.26	0.15 - 1.4	0 - 1.05	0 - 0.42	0 - 0.62	0 - 0.87
	$\delta = 0.1$	Inclusion percentage	96%	94%	90%	89%	100%	87%	84%	86%
		Mean	1.01	2.03	1.45	0.86	0.39	0.15	0.25	0.35
		Median	1.01	1.14	1.14	0.91	0.28	0.09	0.19	0.28
		95% CI bounds	0.88 - 1.15	0.38 - 7.79	0.36 - 3.19	0.17 - 1.4	0 - 1.04	0 - 0.42	0 - 0.62	0 - 0.88
	$\delta = 0.2$	Inclusion percentage	97%	98%	90%	87%	100%	87%	84%	83%
		Mean	1.01	2.06	1.42	0.85	0.44	0.16	0.26	0.40
		Median	1.01	1.18	1.15	0.89	0.34	0.10	0.16	0.30
		95% CI bounds	0.88 - 1.15	0.38 - 7.08	0.4 - 3.15	0.18 - 1.4	0 - 1.13	0 - 0.43	0 - 0.66	0 - 0.98
	$\delta = 0.3$	Inclusion percentage	98%	97%	91%	86%	97%	89%	84%	84%
		Mean	1.01	2.16	1.68	0.74	0.51	0.17	0.28	0.41
		Median	1.01	1.22	1.19	0.86	0.42	0.12	0.20	0.31
		95% CI bounds	0.87 - 1.15	0.16 - 8.71	0.27 - 3.54	0.07 - 1.49	0 - 1.24	0 - 0.44	0 - 0.7	0 - 1.11
	$\delta = 0.4$	Inclusion percentage	99%	99%	90%	81%	95%	88%	87%	86%
		Mean	1.01	2.74	1.50	0.75	0.56	0.20	0.30	0.49
		Median	1.01	1.29	1.21	0.80	0.46	0.19	0.20	0.35
		95% CI bounds	0.86 - 1.16	0.22 - 11.51	0.12 - 3.78	0.05 - 1.54	0 - 1.38	0 - 0.46	0 - 0.75	0 - 1.28
**Meta-model estimated by GMA**[*]	$\delta = 0.5$	Inclusion percentage	99%	98%	88%	79%	96%	84%	83%	89%
		Mean	1.01	2.51	1.62	0.72	0.59	0.22	0.37	0.58
		Median	1.01	1.31	1.20	0.72	0.49	0.23	0.38	0.42
		95% CI bounds	0.84 - 1.18	-0.6 - 11.63	-0.07 - 4.36	-0.24 - 1.68	0 - 1.48	0 - 0.5	0 - 0.8	0 - 1.46
	$\delta = 0.6$	Inclusion percentage	99%	99%	89%	84%	95%	81%	86%	89%
		Mean	1.01	2.66	1.56	0.72	0.65	0.24	0.41	0.70
		Median	1.01	1.21	1.14	0.69	0.54	0.25	0.45	0.56
		95% CI bounds	0.84 - 1.18	-0.16 - 12.4[illegible]	-0.44 - 4.4	-0.24 - 1.78	0 - 1.63	0 - 0.52	0 - 0.87	0 - 1.65
	$\delta = 0.7$	Inclusion percentage	98%	98%	87%	81%	94%	80%	85%	93%
		Mean	1.01	2.91	1.92	0.56	0.67	0.25	0.47	0.84
		Median	1.01	1.16	1.14	0.63	0.47	0.26	0.53	0.68
		95% CI bounds	0.84 - 1.19	-1.61 - 16.8[illegible]	-0.57 - 5.66	-0.72 - 1.82	0 - 1.77	0 - 0.55	0 - 0.97	0 - 1.86
	$\delta = 0.8$	Inclusion percentage	98%	98%	89%	81%	95%	81%	87%	97%
		Mean	1.01	2.92	1.69	0.62	0.70	0.29	0.54	0.89
		Median	1.00	1.23	1.13	0.56	0.59	0.34	0.68	0.64
		95% CI bounds	0.8 - 1.21	-1.93 - 15.8[illegible]	-0.44 - 4.89	-0.4 - 1.76	0 - 1.9	0 - 0.58	0 - 1.04	0 - 2.07
	$\delta = 0.9$	Inclusion percentage	98%	99%	89%	81%	95%	78%	87%	98%
		Mean	1.01	2.42	1.79	0.60	0.71	0.31	0.60	1.07
		Median	1.00	1.03	1.06	0.52	0.53	0.39	0.75	1.12
		95% CI bounds	0.79 - 1.22	-3.63 - 18.1[illegible]	-0.75 - 5.34	-1.19 - 2	0 - 2.04	0 - 0.6	0 - 1.14	0 - 2.28
	$\delta = 1$	Inclusion percentage	98%	99%	89%	78%	93%	73%	88%	97%
		Mean	1.01	2.50	1.81	0.55	0.80	0.33	0.66	1.11
		Median	1.00	1.00	0.95	0.47	0.59	0.41	0.82	1.05
		95% CI bounds	0.78 - 1.23	-10.22 - 19.7	-1.56 - 6.86	-0.43 - 1.97	0 - 2.17	0 - 0.64	0 - 1.23	0 - 2.53

[1] Percentage of estimated 95% confidence intervals including the true value over 100 estimations, for each value of δ;
[2] Mean of 100 estimations;

[3] Median of 100 estimations;
[4] 95% confidence interval estimated using bootstrap method;
[*] Covariance for explanatory variables are estimated using GMA— β_{8-13} are used as the elements of a square root matrix of the covariance matrix. Estimating the square root matrix ensures that the covariance matrix is always semi-definite positive;
[†] We assumed that all three explanatory variables are affected by measurement errors; therefore, after generating explanatory multivariate random variables, we added random measurement errors with standard deviation of β_{5-7}. Also, the true values of β_{5-7} are proportional to δ and the true standard deviations of the explanatory variables ($\sqrt{0.2}$, $\sqrt{1}$, and $\sqrt{5}$).

Table H. Studies which estimate BMR (in Kcal/day) for white males, over 18 years old, based on a recent review of the literature [16]

Study*	Estimated Models (intercept, variable coefficients, and $\sigma(\varepsilon)$ used as signatures)	R^2 (used as signature)	n[1]	Sample Statistics†						Change in goodness of fit	
				W[5]	H[4]	A[3]	L[7]	F[6]	BMI[8]	Δ%[9]	Δ%/#Sgnt. [10]
Lazzer et al., 2010. [48]	$BMR_1 = 1050 + 11W - 3.3A + \varepsilon_1$	0.60	2000	123.9 (22.6)	170 (10)	46.3 (13.8)	78.2 (14.4)	45.8 (8.5)	41.6 (6.8)	0.3%	1.6%
	$BMR_2 = 542 + 10.5W + 3.1H - 3.1A + \varepsilon_2$	0.60								10.2%	
	$BMR_3 = 830 - 2.4A + 19.6L + \varepsilon_3$	0.59								11.0%	
	$BMR_4 = 1021 - 3.3A + 11.9L + 9.8F + \varepsilon_4$	0.60								7.8%	
Bosy-Westphal et al., 2008. [54]	$BMR_5 = 865 - 5.3A + 17.1L + 10.1F + \varepsilon_5$	0.7	69	83 (10.4)	174 (6)	68.8 (6.7)	58.3 (6)	24.6 (8.6)	27.4 (3.4)	0.9%	0.2%
			123	90.7 (15)	181 (7)	45.7 (6.6)	65.7 (8.6)	24.9 (12.4)	27.6 (4.2)		
Huang et al., 2004. [53]	$BMR_6 = 329 + 10W + 4.1H - 2.3A + \varepsilon_6$	0.750	218	146.4 (32.3)	176.1 (8.6)	43.9 (12.9)	81 (12)	62.5 (21.9)	47.1 (9.2) >35	2.2%	1.3%
	$BMR_7 = 1926 - 6.2A + 11.5F + \varepsilon_7$	0.657								2.5%	
	$BMR_8 = 1436 - 6.1A + 26.8BMI + \varepsilon_8$	0.647								7.5%	
	$BMR_9 = 826 - 2.9A + 20.5F + \varepsilon_9$	0.588								9.3%	
Javed et al., 2010. [55]	$BMR_{10} = 793 - 3.1A + 14L + 6.2F + \varepsilon_{10}$	0.696	24	78 (11.5)	177 (8)	51 (21)	62.4 (6.9)	14.8 (8.6)	24.9 (3.3)	1.4%	0.3%
Piers et al., 1998. [50]‡	$BMR_{11} = 764 + 12.2W + \varepsilon_{11}$	0.9	38	73.4 (10.9)	177.2 (7.7)	23 (3)	61.4 (10.2)	12.5 (6.7)	23.1 (3.2)	0.7%	0.2%
	$BMR_{12} = 556 + 12.2W + \varepsilon_{12}$	0.9	24	79.2 (10.8)	176.9 (7.5)	62.3 (8)	58.7 (9.7)	18.6 (7.1)	25.2 (3.4)	0.8%	
Soares et al., 2000. [56]‡	$BMR_{13} = 403 + 19.4L + 8.8F + \varepsilon_{13}$	-	48	76.4 (9.8)	-	24.9 (2.4)	-	-	23.7 (2.3)	0.5%	0.3%
	$BMR_{14} = 267 + 19.4L + 8.8F + \varepsilon_{14}$	-	28	80.2 (10.6)	-	63.3 (5.6)	-	-	26.3 (3.6)	1.3%	
Ravussin et al., 1992. [57]	$BMR_{15} = 914 - 4.3A + 14.3L + 6F + \varepsilon_{15}$	0.68	327	93.2 (39.8)	-	32 (15.8)	-	23.3 (10.7)	-	2.1%	0.4%

Study*	Estimated Models (intercept, variable coefficients, and $\sigma(\varepsilon)$ used as signatures)	R^2 (used as signature)	n[1]	Sample Statistics† W[5]	H[4]	A[3]	L[7]	F[6]	BMI[8]	Change in goodness of fit Δ%[9]	Δ%/#Sgnt. [10]
Tataranni et al., 1995. [58]	$BMR_{16} = 681 - 1.6A + 15.9L + 5.9F + \varepsilon_{16}$	0.73	353	100 (28)	173 (6)	29 (7)	66 (11)	34 (19)	-	0.9%	0.2%
Blanc et al., 2004. [59]	$BMR_{17} = 1294 + 9.7W - 8.6A + \varepsilon_{17}$<br>$BMR_{18} = 773 - 8.6A + 22.8L + \varepsilon_{18}$	0.74<br>0.77	72	85.5 (12.5)	-	75.1 (3.2)	58 (6.1)	25 (7.6)	27.6 (4.2)	0.9%<br>0.6%	0.2%
De Lorenzo et al., 2000. [52]	$BMR_{19} = 841 + 12.8W + \varepsilon_{19}$<br>$BMR_{20} = 1186 + 12.5W - 10.6A + \varepsilon_{20}$	0.34<br>0.69	46	80.1 (10.8)	177.4 (6.8)	30.2 (13.1)	61.8 (8.2)	-	25.4 (2.7)	18.0%<br>11.3%	4.2%
Ferraro et al., 1992. [60]	$BMR_{21} = 754 - 4.5A + 17.2L + 5.3F + \varepsilon_{21}$	0.639	114	84.1 (23.6)	176 (7)	34 (14)	63.2 (10.3)	20.8 (16.5)	-	2.4%	0.5%
Ganpule et al., 2007. [61]	$BMR_{22} = 30 + 11.5W + 5.6H - 3.3A + \varepsilon_{22}$<br>$BMR_{23} = 573 - 2.6A + 18.8L + 6.4F + \varepsilon_{23}$	0.834<br>0.840	71	68.3 (11.5)	170.5 (7.1)	36 (16)	55.3 (7.4)	12.9 (6.4)	23.4 (3.1)	1.5%<br>1.9%	0.3%
Fontvieille et al., 1993. [62]	$BMR_{24} = 767 - 2.2A + 15L + 4.4F + \varepsilon_{24}$	0.88	63	98 (36)	177 (6)	31 (9)	-	-	-	1.0%	0.2%
Nielsen et al., 2000. [63]	$BMR_{25} = 888 - 5.2A + 15.6L + 7.8F + \varepsilon_{25}$	0.45	100	-	-	32 (8)	63.7 (17.7)	15.9 (20.6)	25.1 (9.3)	1.6%	0.3%
Wyatt et al., 1999. [64]‡	$BMR_{26} = 753 - 4.1A + 17.2L + 7.9F + \varepsilon_{26}$	0.805	16	79.8 (12)	178 (7.5)	46.2 (11.2)	58.8 (9.1)	17.3 (8)	25 (3.6)	0.7%	0.1%
Luhrmann et al., 2010. [65]	$BMR_{27} = 63 + 27.5L + \varepsilon_{27}$	0.53	155	79.7 (10.9)	172.9 (6.5)	66.9 (5.2)	58.2 (5.9)	21.4 (6.4)	26.7 (3.4)	0.9%	0.3%

[1]n: sample size; [2]reported values for each measure are mean and (standard deviation); [3]Age (years); [4]Height (cm); [5]Weight (kg); [6]Fat Mass (kg); [7]Lean Mass (kg); [8]Body Mass Index (kg m^{-2}); [9]percentage of reduction in goodness of fit measure when excluding each prior model from calculation of the measure; [10]total Δ% of the study divided by the number of signature of the study, providing a normalized measure of impact of study.

*Studies that do not include the sex effect in regression are excluded. †Standard deviations are reported in the studies, or estimated from standard error of the mean (SEM) or ranges. ‡Studies that mixed sample statistics for females and males—gender differences for the similar sample population in NHANES database are used to estimate sample statistics for males.

Table I. Parameter estimates and standard deviations for coefficients of the four different meta-model specifications

**Hypothesized meta-model**	**MSC**
BMR=558 + 2.8H + 7.5F + 12L - 3.1A + N(0,170)<br>(38)* (0.2) (0.1) (0.1) (0.1) (10)	2,676
BMR=851 + 1.1H + 8.7F + 13L - 3A - 3.3BMI + N(0,172)<br>(48) (0.3) (0.2) (0.2) (0.1) (0.7) (10)	2,722
BMR=231 + 4.4H + 3.1F + 16.2L - 2.4A + $0.06F^2$ - $0.03L^2$ + N(0,128)<br>(121) (0.4) (0.9) (2.4) (0.2) (0.01) (0.02) (10)	2,429
BMR= -3526 + 3.6H + 11F - 5.8L - 2.6A - 130 ln(F) + 1299 ln(L) + N(0,136)<br>(529) (0.4) (0.6) (2.4) (0.15) (20) (161) (11)	**2,390**

***(standard deviation)

Table J. Alternative 95% confidence intervals found using bootstrapping for the BMR estimation

Meta-model 4 in Table I

$$BMR = -3526 + 3.6H + 11F - 5.8L - 2.6A - 130\ln(F) + 1299\ln(L) + N(0,136)$$

(-5084,-1966)*	(2.9,5.2)	(8.6,11.9)	(-12.8,1.9)	(-3,-2.3)	(-160,-47)	(750,1778)	(0,148)

*(95% confidence interval)

Table K. The sampling functions used to generate the required samples of explanatory variables for the underlying studies using the NHANES data.

Study	Models*	Estimated parameters in the sampling function†											
		w						*m*					
		w_1	w_2	w_3	w_4	w_5	w_6	m_1	m_2	m_3	m_4	m_5	m_6
Lazzer et al., 2010. [48]	1,2,3,4	0.53	-0.91	-0.22				67.13	144.34	38.96			
Bosy-Westphal et al., 2008. [54]	5	-0.08	-0.06	-0.21				82.04	177.50	69.30			
		-0.22	0.96	-1.00				87.33	167.50	46.64			
Huang et al., 2004. [53]	6,7,8,9	-0.96	1.00	-0.06				156.60	193.27	43.74			
Javed et al., 2010. [55]	10	-0.88	-0.13	1.00				77.75	177.00	45.00			
Piers et al., 1998. [50]‡	11,12	-0.06	0.03	-0.46				65.41	157.74	23.00			
		-0.07	-0.09	-0.15				72.98	220.91	63.22			
Soares et al., 2000. [56]‡	13,14	0.002		-0.64			-0.47	54.43		25.16			23.25
		-0.07		-0.23			-0.11	82.23		62.33			10.58
Ravussin et al., 1992. [57]	15	0.97		-0.63				79.95		0.02			
Tataranni et al., 1995. [58]	16	-0.06	-0.16	-0.17				185.0	167.00	27.00			
Blanc et al., 2004. [59]	17,18	-0.01		-0.43			0	218.0		75.19			29.60
De Lorenzo et al., 2000. [52]	19,20	0.28	-1.00	-0.48				162.23	181.90	17.20			
Ferraro et al., 1992. [60]	21	1.00	-0.75	-0.56				85.10	177.83	25.69			
Ganpule et al., 2007. [61]	22,23	-0.93	-1.00	1.00				56.43	164.63	71.75			
Fontvieille et al., 1993. [62]	24	0.97	-1.00	-0.69				78.50	174.69	2.13			
Nielsen et al., 2000. [63]	25			-0.48	0.12	-0.22	0.75			33.13	52.33	0.03	27.76
Wyatt et al., 1999. [64]‡	26	-0.22	0.38	-0.28				65.31	158.97	50.25			
Luhrmann et al., 2010. [65]	27	-0.08	-0.04	-0.29				76.26	177.22	66.78			

*See **Table H**. †Probability of data selection is $P(accepting\ a\ data\ point) = (1 + \exp(-(\sum_1^N w_n|m_n - u_n|)))^{-1}$ where *w* and *m* are estimated using the GMA, and *u* is sample statistics representing *W*, *H*, *A*, *L*, and *F* (provided in **Table H**).

Table L. Impact of different measurement technologies

Estimation	Measurement technology	Database/Study	Impact*
Fat Mass	Dual-energy X-ray absorptiometry (DXA)	NHANES; Javed et al., 2010; Piers et al., 1998; Blanc et al., 2004; De Lorenzo et al., 2000; Nielsen et al., 2000; Wyatt et al., 1999.	1
	Siri's equation and BOD-POD for body density	Bosy-Westphal et al., 2008.	0.838 (0.795,0.916)†
	Bioelectrical impedance analysis	Lazzer et al., 2010; Huang et al., 2004; Luhrmann et al., 2010.	1.036 (1.033,1.040)
	Hydostatic	Ravussin et al., 1992; Tataranni et al., 1995; Ferraro et al., 1992; Fontvieille et al., 1993.	0.844 (0.712,1.110)
	Equations based‡	Ganpule et al., 2007.	0.865 (0.708,1.025)
	Total body water	Soares et al., 2000.	0.963 (0.711,1.700)
BMR	Deltatrac	Huang et al., 2004; Javed et al., 2010; Piers et al., 1998; Soares et al., 2000; Blanc et al., 2004; Nielsen et al., 2000; Luhrmann et al., 2010.	1
	Chamber	Ravussin et al., 1992; Ferraro et al., 1992; Ganpule et al., 2007; Fontvieille et al., 1993.	0.932 (0.891,0.990)
	Pneumotachograph	Tataranni et al., 1995.	0.882 (0.846,0.924)
	Vmax 29	Lazzer et al., 2010; Bosy-Westphal et al., 2008.	1.038 (1.028,1.048)
	SensorMedics 2900	Wyatt et al., 1999; De Lorenzo et al., 2000.	0.956 (0.940,0.983)

*Taking Deltatrac and DXA as the standard methods for measuring BMR and Fat Mass, respectively, the impacts of other factors are estimated by GMA. †95% confidence interval estimated using bootstrap method; ‡Tahara's equations (2002) are used to predict body density from the sum of skinfold thickness. Then, Brozek equation (1963) is used for estimation of body fat percentage.

Supplementary Figures

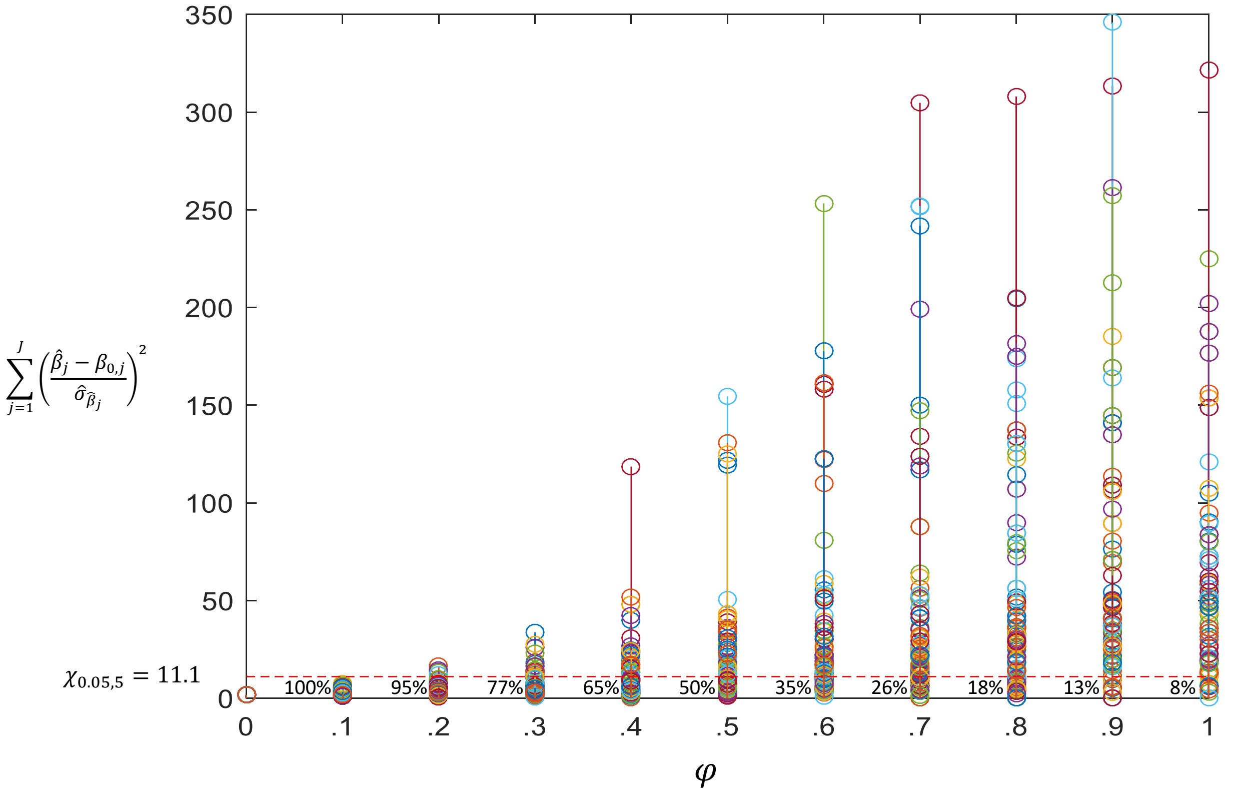

Figure A. Sensitivity of the estimated parameters in scenarios 1 (two independent variable per prior study) to the error (φ) in correlation of the independent variables used for data generation. Normalized squared errors are reported for 100 random correlation matrices for each value of φ.

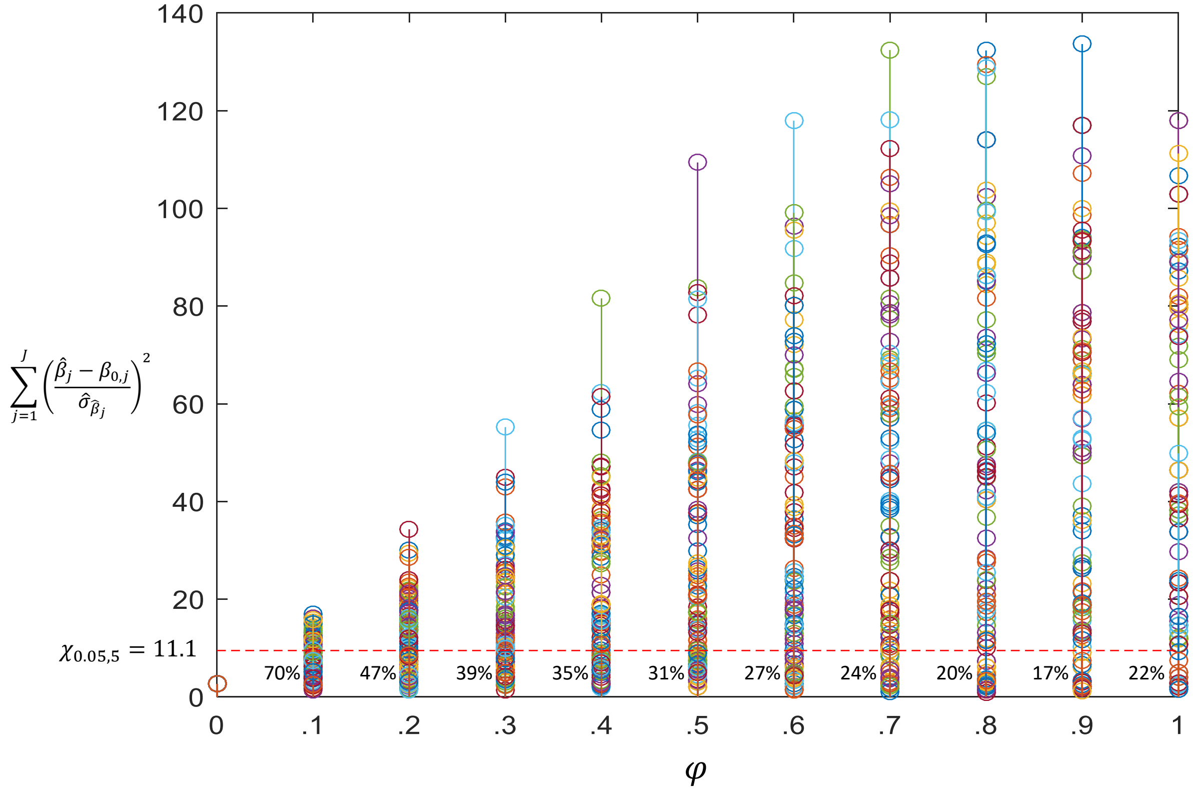

Figure B. Sensitivity of the estimated parameters in 2 (one independent variable per prior study) to the error (φ) in correlation of the independent variables used for data generation. Normalized squared errors are reported for 100 random correlation matrices for each value of φ.

References:

48. Lazzer S, Bedogni G, Lafortuna CL, Marazzi N, Busti C, Galli R, et al. Relationship between basal metabolic rate, gender, age, and body composition in 8,780 white obese subjects. Obesity (Silver Spring). 2010;18(1):71-8. doi: 10.1038/oby.2009.162. PubMed PMID: 19478787.
49. Cai T, Liu W, Xia Y. Two-Sample Covariance Matrix Testing and Support Recovery in High-Dimensional and Sparse Settings. Journal of the American Statistical Association. 2013;108(501):265-77. doi: 10.1080/01621459.2012.758041.
50. Piers LS, Soares MJ, McCormack LM, O'Dea K. Is there evidence for an age-related reduction in metabolic rate? Journal of applied physiology (Bethesda, Md : 1985). 1998;85(6):2196-204. Epub 1998/12/08. PubMed PMID: 9843543.
51. Hendel HW, Gotfredsen A, Hojgaard L, Andersen T, Hilsted J. Change in fat-free mass assessed by bioelectrical impedance, total body potassium and dual energy X-ray absorptiometry during prolonged weight loss. Scandinavian journal of clinical and laboratory investigation. 1996;56(8):671-9. Epub 1996/12/01. PubMed PMID: 9034348.
52. De Lorenzo A, Andreoli A, Bertoli S, Testolin G, Oriani G, Deurenberg P. Resting metabolic rate in Italians: relation with body composition and anthropometric parameters. Acta Diabetol. 2000;37(2):77-81. PubMed PMID: 11194931.
53. Huang KC, Kormas N, Steinbeck K, Loughnan G, Caterson ID. Resting metabolic rate in severely obese diabetic and nondiabetic subjects. Obes Res. 2004;12(5):840-5. doi: 10.1038/oby.2004.101. PubMed PMID: 15166305.
54. Bosy-Westphal A, Wolf A, Buhrens F, Hitze B, Czech N, Monig H, et al. Familial influences and obesity-associated metabolic risk factors contribute to the variation in resting energy expenditure: the Kiel Obesity Prevention Study. Am J Clin Nutr. 2008;87(6):1695-701. Epub 2008/06/11. PubMed PMID: 18541558.
55. Javed F, He Q, Davidson LE, Thornton JC, Albu J, Boxt L, et al. Brain and high metabolic rate organ mass: contributions to resting energy expenditure beyond fat-free mass. Am J Clin Nutr. 2010;91(4):907-12. doi: 10.3945/ajcn.2009.28512. PubMed PMID: 20164308; PubMed Central PMCID: PMC2844678.
56. Soares MJ, Piers LS, O'Dea K, Collier GR. Plasma leptin concentrations, basal metabolic rates and respiratory quotients in young and older adults. Int J Obes Relat Metab Disord. 2000;24(12):1592-9. doi: 10.1038/sj.ijo.0801450. PubMed PMID: 11126211.
57. Ravussin E, Rising R. Daily energy-expenditure in humans - measurements in a respiratory chamber and by doubly labeled water. Kinney JM, Tucker HN, editors1992. 81-96 p.
58. Tataranni PA, Ravussin E. Variability in metabolic rate: biological sites of regulation. Int J Obes Relat Metab Disord. 1995;19 Suppl 4:S102-6. PubMed PMID: 8581083.
59. Blanc S, Schoeller DA, Bauer D, Danielson ME, Tylavsky F, Simonsick EM, et al. Energy requirements in the eighth decade of life. Am J Clin Nutr. 2004;79(2):303-10. PubMed PMID: 14749238.
60. Ferraro R, Lillioja S, Fontvieille AM, Rising R, Bogardus C, Ravussin E. Lower sedentary metabolic rate in women compared with men. The Journal of clinical investigation. 1992;90(3):780-4. doi: 10.1172/JCI115951. PubMed PMID: 1522233; PubMed Central PMCID: PMC329930.
61. Ganpule AA, Tanaka S, Ishikawa-Takata K, Tabata I. Interindividual variability in sleeping metabolic rate in Japanese subjects. Eur J Clin Nutr. 2007;61(11):1256-61. doi: 10.1038/sj.ejcn.1602645. PubMed PMID: 17299487.
62. Fontvieille AM, Ferraro RT, Rising R, Larson DE, Ravussin E. Energy cost of arousal: effect of sex, race and obesity. Int J Obes Relat Metab Disord. 1993;17(12):705-9. PubMed PMID: 8118475.
63. Nielsen S, Hensrud DD, Romanski S, Levine JA, Burguera B, Jensen MD. Body composition and resting energy expenditure in humans: role of fat, fat-free mass and extracellular fluid. Int J Obes Relat Metab Disord. 2000;24(9):1153-7. doi: 10.1038/sj.ijo.0801317. PubMed PMID: 11033984.

64. Wyatt HR, Grunwald GK, Seagle HM, Klem ML, McGuire MT, Wing RR, et al. Resting energy expenditure in reduced-obese subjects in the National Weight Control Registry. Am J Clin Nutr. 1999;69(6):1189-93. PubMed PMID: 10357738.
65. Luhrmann PM, Edelmann-Schafer B, Neuhauser-Berthold M. Changes in resting metabolic rate in an elderly German population: cross-sectional and longitudinal data. The journal of nutrition, health & aging. 2010;14(3):232-6. Epub 2010/03/02. PubMed PMID: 20191259
