# Supplementary material for: A flexible method for aggregation of prior statistical findings
Source: PLoS One. 2017 Apr 6;12(4):e0175111. doi: 10.1371/journal.pone.0175111 (PMC5383132; doi:10.1371/journal.pone.0175111)
Supplement: S2 File — This document presents: (1) instructions on how to read and execute the codes, (2) descriptions about the notations and functions in the codes, and (3) details of the codes. (PDF) [file pone.0175111.s002.pdf]

README for Generalized Model Aggregation (GMA) codes

MATLAB version

Summary:

This document provides:

- instructions on how to read and execute the codes
- descriptions about the notations and functions in the codes
- details of the codes

Contents:

I. Instructions 2

General overview 2

Organization of the codes 2

How to read the codes 2

How to execute the codes 2

The current codes 2

How to modify the codes for your research project 2

II. Notations in MATLAB codes 3

Table 1: Notations 3

III. MATLAB functions 4

Table 2: User-specified files 4

Table 3: GMA-specific files 5

IV. MATLAB Codes 6

I. Instructions

General overview

The codes in this document are developed using MATLAB (a matrix-based and proprietary language, available at http://www.mathworks.com). The codes are written in 14 '.m' files and included in a zipped file (supplementary **S3 File**), attached to this electronic companion.

Organization of the codes

The 14 .'m' files are divided into two general groups: user-specified and GMA-specific files. The user-specified files should be filled out based on information of your aggregation project; however, the GMA-specific files remain the same. **Table 2** and **Table 3** present user-specified and GMA-specific files, respectively, and provide general information about their action, inputs, and outputs.

How to read the codes

Table 1 provides the list of nations in the codes along with their definitions. If you do not have MATLAB on your machine, you can open '.m' files in any text editor—however, you need to have MATLAB installed in order to execute the codes. For your convenience, the codes are also provided in Section **IV. MATLAB Codes** of this document.

How to execute the codes

To run the codes, open *GMA_RUN.m* in MATAB Editor and execute the codes (click on 'Run' button on the top toolbar in MATLAB Editor, or write `run('GMA_RUN.m')` in MATLAB Command Window).

Example embedded in the current codes

The current codes present Scenario 1 in the paper, where the "true" data generating process is $\mathbf{y} = 1 + \mathbf{x}_1 + \mathbf{x}_2 + \mathbf{x}_3 + \boldsymbol{\varepsilon}$ (the coefficient of the three explanatory variables and the standard deviation of the error term are assumed to be one). Regression results from three "prior" studies, all assumed to be linear regressions, are considered (see three User_model files). Each imitated prior study uses a constant and two of the three explanatory variables. Hence, the constant, two coefficient of the explanatory variables, and standard deviation of the error term provide four empirical signatures for each model (therefore, there are totally 3*4=12 signatures in this example).

How to modify the codes for your research project

To modify the codes, you should complete the User-specified files. The major work here is to complete the files for two categories in **Table 2**: '**Replication of prior models**' and '**Generating simulated data for explanatory and response variables**'. See the paper for more information.

II. Notations in MATLAB codes

Table 1: Notations

**Notation** Sorted alphabetically	**Definition**
Beta	The parameters of the meta-model
Beta_int	Starting points for the parameters of the meta-model (Beta) to initiate the optimization search
ConfDown	Lower confidence interval of the estimated Beta
ConfLvl	Confidence level (e.g., 95%) for estimation of the confidence interval of Beta
ConfUp	Upper confidence interval of the estimated Beta
E	The difference between empirical and simulated signatures
EmpSign	Empirical signatures extracted from prior studies
Error	Random error term in the meta model
J	Number of parameters in Beta (the size of vector Beta)
L (and l)	The number of prior studies to aggregate; l ∈ {1,..,L}
mu	Mean of each explanatory variable, used in generating multivariate explanatory variables (X)
n	Sample size in a prior study
Opt_lb	Lower bound for Beta used by the optimization solver, if needed be (e.g., for a parameter that is always positive such as standard deviation)
Opt_method	Optimization method, e.g., global search (GS) or multi-start (MS) search. See MATLAB help for more information.
Opt_MS_inst	The number of instances of start points, used 'only' if multi-start (MS) optimization search is selected.
Opt_n	The number of iterations to estimate W and then run the optimization with the new W
Opt_Tolrnc	Tolerance (threshold), a stopping criterion for the optimization solver
Opt_ub	Upper bound for Beta, if needed be, used by the optimization solver
Q	Covariance matrix of the estimated Beta
r	The number of iterations used only for bootstrapping—if no bootstrap is used, *r* should be 1. See the paper for more information on bootstrapping.
S (and s)	The number of simulations (replications) in equation $\mathbf{e}_l\left(\tilde{\boldsymbol{\gamma}}_l, \mathbf{X}_l^s, \mathbf{y}_l^s(\boldsymbol{\beta})\right) = \frac{1}{S}\sum_{s=1}^{S}\left(\tilde{\boldsymbol{\gamma}}_l - \boldsymbol{\gamma}_l^s(\boldsymbol{\beta})\right)$; s ∈ {1,..,S}
S_large	The number of simulations but larger than S to better estimate the weighting matrix (W)
SIGMA	Covariance matrix of the explanatory variables (X)
SimSign	Simulated signatures of prior studies
SimSign_S	S vectors of simulated signatures. Average of all these vectors over S would be SimSign.
SS	Covariance matrix of the simulated signatures. The inverse of SS would be W.
Std_Beta	Standard deviation of the estimated Beta
SX	Sample from X for a prior study. SX could include a subset of variables in X based on the data used in the respective prior study.
W	Weighting matrix: when Opt_n=1, W=diag(1./(EmpSign.^2)); when Opt_n>1, W is the output of function *GMA_W_star.m* (see *GMA_RUN.m*).
X	Simulated multivariate explanatory variables
Y	Simulated response variable calculated by the meta model

III. MATLAB functions

The files in **Table 2** should be completed by the user based on the aggregation research project. The files in **Table 3**, however, are GMA-specific and remain the same for any project.

Table 2: User-specified files

Category	File<br>Click on file names to see the codes.	Action	Function input	Function output
Choose signatures and replicate prior models	*User_model_(l).m*	It first asks users for information from prior study l (i.e., empirical signatures and sample size of the study).<br>It then generates X and Y(Beta), and replicates study l.<br>Finally, the outputs are empirical signatures and estimated simulated signature of study l.	Beta, s, r	EmpSign, SimSign
Generate simulated data for explanatory and response variables	*User_DataGeneration.m*	Generates simulated multivariate explanatory data (X)	SIGMA, n, s, r	X
	User_Meta_model.m	Based on the simulated explanatory data (X) and current Beta, the meta model generates simulated response variable (Y).	Beta, X, s, r	Y
	User_SIGMA.m	To identify or estimate the covariance matrix of the explanatory variables (X)	-	SIGMA
Initiate GMA and optimization	*User_GeneralInputs.m*	To identify general constants used in GMA (listed constants in column 'Function output')	-	L, S, S_large, Opt_n, r, ConfLvl
	User_OptInitiation.m	To identify an optimization method and pass initial optimization conditions to *Optimization.m*	-	Beta_int, Opt_lb, Opt_ub, Opt_Tolrnc, Opt_method, Opt_MS_inst

Table 3: GMA-specific files

Category	File<br>Click on file names to see the codes.	Action	Function input	Function output
Main script to run GMA	*GMA_RUN.m*	Runs and follows the GMA steps:<br>[1] Calls functions to ask user inputs<br>[2] Estimates weighting matrix (W) and run optimization to estimate Beta. Iterates this step based on Opt_n.<br>[3] Estimates confidence intervals of the estimated Beta<br>Finally, saves the MATLAB workspace.	-	Unlike all other *.m* files here, *GMA_RUN.m* is not a function; however, it saves MATLAB workspace (including estimation results) in a '*.mat*' file.<br>See the 'save' command at the end of the script.
Simulation and optimization	*GMA_Signatures.m*	Captures empirical signatures and replicate simulated signatures from each of the L prior models.	Beta, S, r	EmpSign, SimSign_S
	GMA_Optimization.m	Calls on GMA_ObjFn to estimate Beta. Depending on user inputs specified in *User_OptInitiation.m*, it uses either global-start or multi-start algorithm (see MATLAB help for more information).	Beta_int, W	Beta, ObjFnVal
	GMA_ObjFn.m	Calls for simulated signature based on the current Beta and W, and then provides the value of objective function to pass into *GMA_Optimization.m*.	Beta, W	ObjFnVal
	GMA_W_star.m	Calculates the covariance matrix of the simulated signatures (SS) and the weighting matrix (W)—the inverse of SS is W.	Beta, r	W, SS
Estimation of the variations of the estimated Beta	*GMA_Var_EstimatedVar.m*	Estimates the covariance matrix of Beta (Q).<br>Calls on *GMA_Var_Delta.m* to estimate numerical derivative (i.e., changes in simulated signatures with respect to changes in Beta), and then estimates Q.	Beta, W, SS	Q
	GMA_Var_Delta.m	Estimates the sensitivity of the simulated signatures to the perturbations in Beta.	Beta, J	D
	GMA_Var_ConfInt.m	Calculates confidence intervals of the estimated parameters based on a ConfLvl (e.g., at ConfLvl = 95%).	B, ConfLvl, Std_Beta	ConfDown, ConfUp

IV. MATLAB Codes

For more infromation about the aggregation example presented in the codes, see Section '**Example embedded in the current codes**'.
Codes are published in Microsoft Word format via MATLAB® R2015b publisher.

1. User_model_(l).m 7
2. User_DataGeneration.m 8
3. User_Meta_model.m 9
4. User_SIGMA.m 10
5. User_GeneralInputs.m 11
6. User_OptInitiation.m 12
7. GMA_RUN.m 13
8. GMA_Signatures.m 15
9. GMA_Optimization.m 16
10. GMA_ObjFn.m 17
11. GMA_W_star.m 18
12. GMA_Var_EstimatedVar.m 19
13. GMA_Var_Delta.m 20
14. GMA_Var_ConfInt.m 21

1. User_model_(I).m

```
% Empirical and simulated signatures for prior model 1

function [EmpSign,SimSign]=User_model_1(Beta,s,r)
```

Empirical signatures

this information should be captured from the reported results for this model

```
EmpSign=[0.978; 1.860; 2.226;  2.900];
% in this example, the first three numbers are coefficients of the
% regression and the last number is the error variance of the regression.

n=100; % sample size for this study
```

Simulated signatures

1) Generate simulated explanatory variables (X)

```
SIGMA=User_SIGMA(); % Covariance matrix of X
X=User_DataGeneration(SIGMA,n,s,r); % X is generated in User_DataGeneration function

% 2) Generate simulated Y using the meta model, simulated X, and Beta
Y=User_Meta_model(Beta,X,s,r);

% 3) Sample from X (based on the data used in this model)
SX = [ones(n,1) X(:,1) X(:,2)]; % In this example, X has three variables (X1-X3) but
% this study only uses two of the variables

% 4) Calculate the signatures
% Empirical signatures in this studies were results of regression modeling,
% therefore regression is run on Y and SX to estimate simulated signatures.
[coeff,~,~,~,StatResults] = regress(Y,SX);
ErrVar=StatResults(4);

SimSign=[coeff; ErrVar]; % put all signatures in a vector of simulated signatures
% >> IMPORTANT: SimSign must be sorted based on their empirical counterparts (EmpSign).

end
```

2. User_DataGeneration.m

```
% To generate simulated explanatory variables (X)

function [X]=User_DataGeneration(SIGMA,n,s,r)

mu=[0,0,0]; % mean of each variable. In this example, there are three variables, X1-X3
```

multivariate random data generation (X)

```
rng(s+r*2000) % to control the random generation process (i.e., fix the noise seed
% based on s (number of simulations) and r (number of replications for bootstrapping).

X = mvnrnd(mu,SIGMA,n); % explanatory variables; multivariate normal random variables

end
```

3. User_Meta_model.m

```
function Y=User_Meta_model(Beta,X,s,r)
```

```
n=size(X,1); % sample size of the simulated explanatory variables (X)
```

Error term in the meta model

```
rng(s+r*1000) % to control the random generation process (i.e., fix the noise seed
% based on s (number of simulations) and r (number of replications for bootstrapping).
Error= Beta(end)*randn(n, 1); % = mean + Std*randn();  where mean=0
% Note that the last value of Beta (Beta(end)) is used as standard deviation for error
% term. See 'User_OptInitioation' function for more info.
```

Meta-model to calculate Y

structure of hypothesized meta-model

```
Y = Beta(1) + Beta(2)*X(:,1)  + Beta(3)*X(:,2) + Beta(4)*X(:,3) + Error;
```

```
end
```

4. User_SIGMA.m

```
function SIGMA=User_SIGMA()

% SIGMA is needed in order to generate random variables X.
% It can be obtained from existing empirical data sets, from prior studies,
% or if there is no information available it can be considered as unknown
% parameters (i.e., as additional components of vector Beta) and be estimated,

% In this example, variance and correlation matrix are obtained from prior studies,
% and the covariance matrix is estimated as follow:
AllVar =[0.21  1.12  4.38];

SDev=sqrt(AllVar);

Corr=[1 0.05 0.2
    0.05 1 0.7
    0.2 0.7 1];

SIGMA=Corr.*(SDev'*SDev);

if sign(eig(SIGMA))<0  %check the positivity of Eigenvalues
    display('**** Note: Covariance matrix is NOT positive semi-definite.')
    beep
end

end
```

5. User_GeneralInputs.m

```
% To be completed by the user; includes general information (i.e.,
% constants such as the number of replications

function [L, S, S_large, Opt_n, r, ConfLvl]=User_GeneralInputs()

L=3; % number of prior models to aggregate
S = 7; % number of simulations (replications)
S_large = 200; % number of simulations, larger than S to better estimate
% the weighting matrix (W)

Opt_n=3;
% When Opt=1, the diagonal of W is the inverse of empirical signatures.
% When Opt=2 or higher, W is the inverse of covariance matrix of simulated
% signatures, given the estimated Beta.
% Each new W is used for the next round of Beta estimation.
% This process can be continued until convergency is achieved, however most
% problems typically achieve convergency in a few iterations.
% Opt should be always >=1.

r=1; % an arbitrary parameter in the codes representing the number of iterations
% for bootstrapping. It changes the noise seed of random generation processes in
% the codes. r is only used for bootstrapping when it should be larger than
% one (see Supplementary Notes on Bootstrapping).

ConfLvl=0.95; % Confidence level (e.g., 95%), used in the estimation of
% confidence interval of Beta

end
```

6. User_OptInitiation.m

```
% To be completed by the user; includes information to run the optimization search

function [Beta_int,Opt_lb,Opt_ub,Opt_Tolrnc,Opt_method,Opt_MS_inst]=User_OptInitiation()

Beta_int = [0.5 0.5 0.5 0.5 0.5 ];  % start points for the optimization search
% see 'User_Meta_Model' for the structure of meta model and how Beta_int is used there.

% lb: lower bound for Beta used by the optimization solver.
% In this example, the last number represents standard deviation which
% should be a positive number.
Opt_lb = [-inf -inf -inf -inf 0  ];
% ub: upper bound for Beta
Opt_ub = [ inf  inf  inf  inf inf ];

Opt_Tolrnc = 1e-6;
% Tolerance, used by the optimization solver, is a threshold which,
% if crossed, the iterations of the optimization solver are stopped.

Opt_method='MS'; % 'MS' or 'GS'
% MS: Multi-start.
% GS: Global search. See MATLAB help for more information on MS and GS.

Opt_MS_inst=1; % If 'MS' is selected, the number of instances of start pints
% should be specified.
end
```

7. GMA_RUN.m

```
clc; tic
```

Inputs from user

```
[~,S,~,Opt_n,r,ConfLvl]=User_GeneralInputs(); % to call parameters for running the GMA.
% See the function (User_GeneralInputs) for more information.
Beta_int=User_OptInitiation(); % to call start points for the optimization search
```

Estimate weighting matrix (W) and run optimization

```
for z=1:Opt_n
    if z==1
        % First round of optimization based on initial W
        [EmpSign,~]=GMA_Signatures(Beta_int,1,r); % to call empirical signatures
        W=diag(1./(EmpSign.^2)); % the diagonal of this W is the inverse of
        % empirical signatures.
        [Beta,ObjFnVal]=GMA_Optimization(Beta_int,W); % to run optimization solver
        % optimization search starts from Beta_int and its output, estimated
        % parameters are Beta.
    else
        % Additional rounds of optimization based on updated W
        [W,SS]=GMA_W_star(Beta,r); % here W is the inverse of covariance matrix
        % of simulated signatures, given the estimated Beta.
        [Beta,ObjFnVal]=GMA_Optimization(Beta,W); % to run optimization solver
        % optimization search starts from Beta
    end
    % capture estimated W, Beta, and ObjFnVal in each iteration (not needed
    % in GMA, captured only for additional analysis if needed be).
    W_all(:,:,z)=W; Beta_all(z,:)=Beta; ObjFnVal_all(z,:)=ObjFnVal;
end
```

Estimate confidence intervals

```
Q = GMA_Var_EstimatedVar(Beta, W, SS); % to calculate the covariance matrix of
% the estimated parameters (Beta).
Std_Beta=sqrt(diag(Q)); % The diagonal of Q includes variances of the estimated Beta
[ConfDown, ConfUp]=GMA_Var_ConfInt(Beta, ConfLvl, Std_Beta); % confidence intervals of
% the estimated Beta

% Wrap up a few additional results (for further analysis):
```

```
SimSign_S=GMA_Signatures(Beta,S,r);
SimSign=sum(SimSign_S, 2)/S; % simulated signatures based on estimated parameters
All_Sign=[EmpSign,SimSign]; % to put empirical and simulated signatures in two
% columns next to each other
```

Save MATLAB workspace

```
save(['Final_Results_Run_' num2str(r) '.mat']); % Use your preferred name
% for this workspace.
display('Done! All results are saved.');
toc; % tic and toc show the total calculation time
```

8. GMA_Signatures.m

```
% to capture empirical and simulated signatures from 'User_Model_(l)' functions

function [EmpSign,SimSign_S]=GMA_Signatures(Beta,S,r)

[L,~,~,~,~,~]=User_GeneralInputs(); % to call the number of prior studies (L)

for s=1:S

    EmpSign=[];
    SimSign=[];

    for l=1:L % Call 'User_Model_(l)' functions where l is 1 to L
        Fn=str2func(['@(Beta,s,r)' 'User_model_' num2str(l) '(Beta,s,r)']);
        [EmpSign_model, SimSign_model]=Fn(Beta,s,r);

        EmpSign=[EmpSign; EmpSign_model];
        % Put all empirical signatures in one vector (EmpSign)
        SimSign=[SimSign; SimSign_model];
        % Put all simulated signatures in one vector (SimSign)
    end

    SimSign_S(:,s)=SimSign;
end
end
```

9. GMA_Optimization.m

```
function [Beta,ObjFnVal]=GMA_Optimization(Beta_int,W)
```

```
[~,Opt_lb,Opt_ub,Opt_Tolrnc,Opt_method,Opt_MS_inst]=User_OptInitiation(); % to call user inputs for the optimization solver

display('******************************');
display('*** REPORT: Optimization begins...');
```

Optimization properties

```
options = optimset;
options = optimset(options,'Display', 'iter-detailed');
options = optimset(options,'TolFun', Opt_Tolrnc);
options = optimset(options,'TolX', Opt_Tolrnc);
options = optimset(options,'PlotFcns', {  @optimplotx @optimplotfunccount @optimplotfval @optimplotstepsize });
options = optimset(options,'Algorithm', 'interior-point');
```

Optimization method, 'GMA_ObjFn' is the obj fn

create an optimization problem

```
problem = createOptimProblem('fmincon','objective',@(Beta)GMA_ObjFn(Beta,W),'x0',Beta_int,'lb',Opt_lb,'ub',Opt_ub,'options',options);

% Run global search or multi start search. See Matlab help for more information.
if strcmp(Opt_method,'GS')
    gs=GlobalSearch('StartPointsToRun','bounds', 'TolFun', Opt_Tolrnc, 'TolX', Opt_Tolrnc);
    [Beta,ObjFnVal] = run(gs,problem);
else
    ms=MultiStart( 'StartPointsToRun', 'bounds', 'TolFun', Opt_Tolrnc, 'TolX', Opt_Tolrnc);
    [Beta,ObjFnVal] = run(ms,problem,Opt_MS_inst);
end

display('*** REPORT: Optimization ends!');
display(Beta);
display('************************************');
```

```
end
```

10. GMA_ObjFn.m

```
function ObjFnVal=GMA_ObjFn(Beta, W)
```

```
[~,S,~,~,r,~]=User_GeneralInputs();

% Call empirical signatures and simulated signatures based on Beta:
[EmpSign, SimSign_S]=GMA_Signatures(Beta,S,r);
SimSign=sum(SimSign_S, 2)/S;
```

Objective function

```
E = EmpSign - SimSign; % The difference between empirical and simulated signatures
ObjFnVal=E' * W * E ; % Objective function used by the optimization solver
```

```
end
```

11. GMA_W_star.m

```
% To estimated weighting matrix W based on estimated parameter (Beta)

function [W,SS]=GMA_W_star(Beta,r)

[~, ~,S_large,~,~,~]=User_GeneralInputs();

% simulation based on the estimated Beat
[~,SimSig]=GMA_Signatures(Beta,S_large,r); % S_large is the number of
% Simulations (larger than S, to better estimate the covariance matrix).

SS=cov(SimSig'); % SS: covariance matrix of the simulated signatures
W=inv(SS);  % Inverse of SS

end
```

12. GMA_Var_EstimatedVar.m

```
function Q = GMA_Var_EstimatedVar(Beta, W, SS)
```

```
[~, S,~,~,~,~]=User_GeneralInputs();
J=size(Beta,2);   % number of parameters
```

Numerical differentiation

changes in simulated signatures with respect to changes in parameters

```
D=GMA_Var_Delta(Beta, J);
```

Estimation of the variations of Beta

```
DW   =  D' * W * D;
DW_1 =  inv(DW);

WDS   = (W * D)' * SS * (W * D);
Q   = (1+1/S) .* DW_1' * (WDS\DW); % Covariance matrix of parameters (Beta).
% The diagonal of this matrix presents the variances of estimated parameters (Beta)
```

```
end
```

13. GMA_Var_Delta.m

```
function D = GMA_Var_Delta(Beta, J)
```

```
eps = 0.001;
[~,S,~,~,r,~]=User_GeneralInputs();
```

Shift parameters (Beta) one epsilon up and down

```
for i=1:J
    for z=1:2*J
        if fix((z+1)/2)==i
            BS(i,z)=Beta(i)*(1+ (rem(z,2)*2-1) * eps);
        else
            BS(i,z)=Beta(i);
        end
    end
end
for i=1:2*J
    [~,Sim_sign]=GMA_Signatures(BS(:,i), S, r);
    AveSim_sign(:,i)=sum(Sim_sign, 2)/S;
end
```

D is used to estimate the changes in simulated signatures with respect to changes in Beta.

```
for i=1:2*J
    if rem(i,2)==0

        LSim(:,fix(i/2))=AveSim_sign(:,i); %  +eps
    else
        USim(:,fix(i/2+0.5))=AveSim_sign(:,i); %  -eps
    end
end
```

```
a=Beta*(1+eps); b=Beta*(1-eps);
sm=size(AveSim_sign, 1);
for i=1:sm
    D(i,:)=(USim(i,:)-LSim(i,:)) ./ (a-b);  % size: m*J, where
    % m is the number of signatures and J is the number of parameters in
    % Beta
end
```

```
end
```

14. GMA_Var_ConfInt.m

```
function [ConfDown, ConfUp]=GMA_Var_ConfInt(Beta, ConfLvl, Std_Beta)

ConfFactor=norminv(1-(1-ConfLvl)/2) ; % based on Student-t

% Upper and lower confidence intervals
ConfDown = Beta' - ConfFactor * Std_Beta;
ConfUp   = Beta' + ConfFactor * Std_Beta;

end
```
